# Performance criteria for verbal autopsy-based systems to estimate national causes of death: development and application to the Indian Million Death Study

**DOI:** 10.1186/1741-7015-12-21

**Published:** 2014-02-04

**Authors:** Lukasz Aleksandrowicz, Varun Malhotra, Rajesh Dikshit, Prakash C Gupta, Rajesh Kumar, Jay Sheth, Suresh Kumar Rathi, Wilson Suraweera, Pierre Miasnikof, Raju Jotkar, Dhirendra Sinha, Shally Awasthi, Prakash Bhatia, Prabhat Jha

**Affiliations:** 1 Centre for Global Heath Research, St. Michael’s Hospital, Dalla Lana School of Public Health, University of Toronto, Toronto, Canada; 2 Tata Memorial Hospital, Mumbai, India; 3 Healis-Sekhsaria Institute for Public Health, Navi Mumbai, India; 4 School of Public Health, Post Graduate Institute of Medical Education and Research, Chandigarh, India; 5 Department of Preventative and Social Medicine, NHL Municipal Medical College, Ahmedabad, India; 6 Rajiv Gandhi Jeevanayee Aarogya Yojana Society, Government of Maharashtra, Mumbai, India; 7 World Health Organization, South-East Asia Regional Office, New Delhi, India; 8 Department of Paediatrics, King George’s Medical University, Lucknow, India; 9 Apollo Institute of Medical Sciences and Research, Hyderabad, India

**Keywords:** Verbal autopsy, Physician-certified verbal autopsy, Cause of death statistics, Vital statistics, India

## Abstract

**Background:**

Verbal autopsy (VA) has been proposed to determine the cause of death (COD) distributions in settings where most deaths occur without medical attention or certification. We develop performance criteria for VA-based COD systems and apply these to the Registrar General of India’s ongoing, nationally-representative Indian Million Death Study (MDS).

**Methods:**

Performance criteria include a low ill-defined proportion of deaths before old age; reproducibility, including consistency of COD distributions with independent resampling; differences in COD distribution of hospital, home, urban or rural deaths; age-, sex- and time-specific plausibility of specific diseases; stability and repeatability of dual physician coding; and the ability of the mortality classification system to capture a wide range of conditions.

**Results:**

The introduction of the MDS in India reduced the proportion of ill-defined deaths before age 70 years from 13% to 4%. The cause-specific mortality fractions (CSMFs) at ages 5 to 69 years for independently resampled deaths and the MDS were very similar across 19 disease categories. By contrast, CSMFs at these ages differed between hospital and home deaths and between urban and rural deaths. Thus, reliance mostly on urban or hospital data can distort national estimates of CODs. Age-, sex- and time-specific patterns for various diseases were plausible. Initial physician agreement on COD occurred about two-thirds of the time. The MDS COD classification system was able to capture more eligible records than alternative classification systems. By these metrics, the Indian MDS performs well for deaths prior to age 70 years. The key implication for low- and middle-income countries where medical certification of death remains uncommon is to implement COD surveys that randomly sample all deaths, use simple but high-quality field work with built-in resampling, and use electronic rather than paper systems to expedite field work and coding.

**Conclusions:**

Simple criteria can evaluate the performance of VA-based COD systems. Despite the misclassification of VA, the MDS demonstrates that national surveys of CODs using VA are an order of magnitude better than the limited COD data previously available.

## Background

Most of the nine million annual deaths in India, as in most low- and middle-income countries (LMICs), occur at home, without medical attention or certification [1-5]. Thus, alternative systems to measure the causes of death (CODs) are needed. Since 2002, the Registrar General of India (RGI) has integrated an enhanced form of verbal autopsy (VA) into its ongoing large-scale, nationally-representative Sample Registration System (SRS), which monitors births and deaths in about one million randomly selected homes [6,7]. Field records are coded by physicians to the International Classification of Diseases (ICD-10) [8]. The main objective of the Million Death Study (MDS) is to reliably document the major CODs in India, their key risk factors and their variation by age, sex and state. To date, about 200,000 records have been double-coded by physicians. The MDS design and methods [6], and results for priority diseases [9-19] and risk factors (most importantly smoking [20]), have already been published. The entire MDS has been done at low cost, at less than $2/household/year [6].

VA-based methods have been widely used in small, focused studies to determine CODs for children, maternal deaths, and more recently, for adults [1-3]. They have increasingly been recommended to determine national COD distributions [1-5], which requires VA on a random sample of deaths. VA-based systems to establish national COD distributions do not yet have performance metrics comparable to those developed for vital registration with medical certification [3,21,22]. Here, we propose simple, replicable performance criteria for VA-based COD systems and apply these to the ongoing MDS. We examine the metrics of ill-defined deaths before age 70 years; reproducibility of COD distributions with independent resampling; differences in COD distributions between hospital versus home deaths, and between urban and rural deaths; age-, sex- and time-specific plausibility of selected diseases; stability and reproducibility of dual physician coding; and finally, the ability of COD classification systems to capture a wide range of conditions in the ICD-10.

We conclude by discussing the lessons learned from the first phase of the MDS, the implications of the MDS for other countries considering VA-based COD systems and provide recommendations to enable efficient and reliable use of VA to determine national COD distributions in other LMICs.

## Methods

### Overview of the Million Death Study

**Figure 1:**
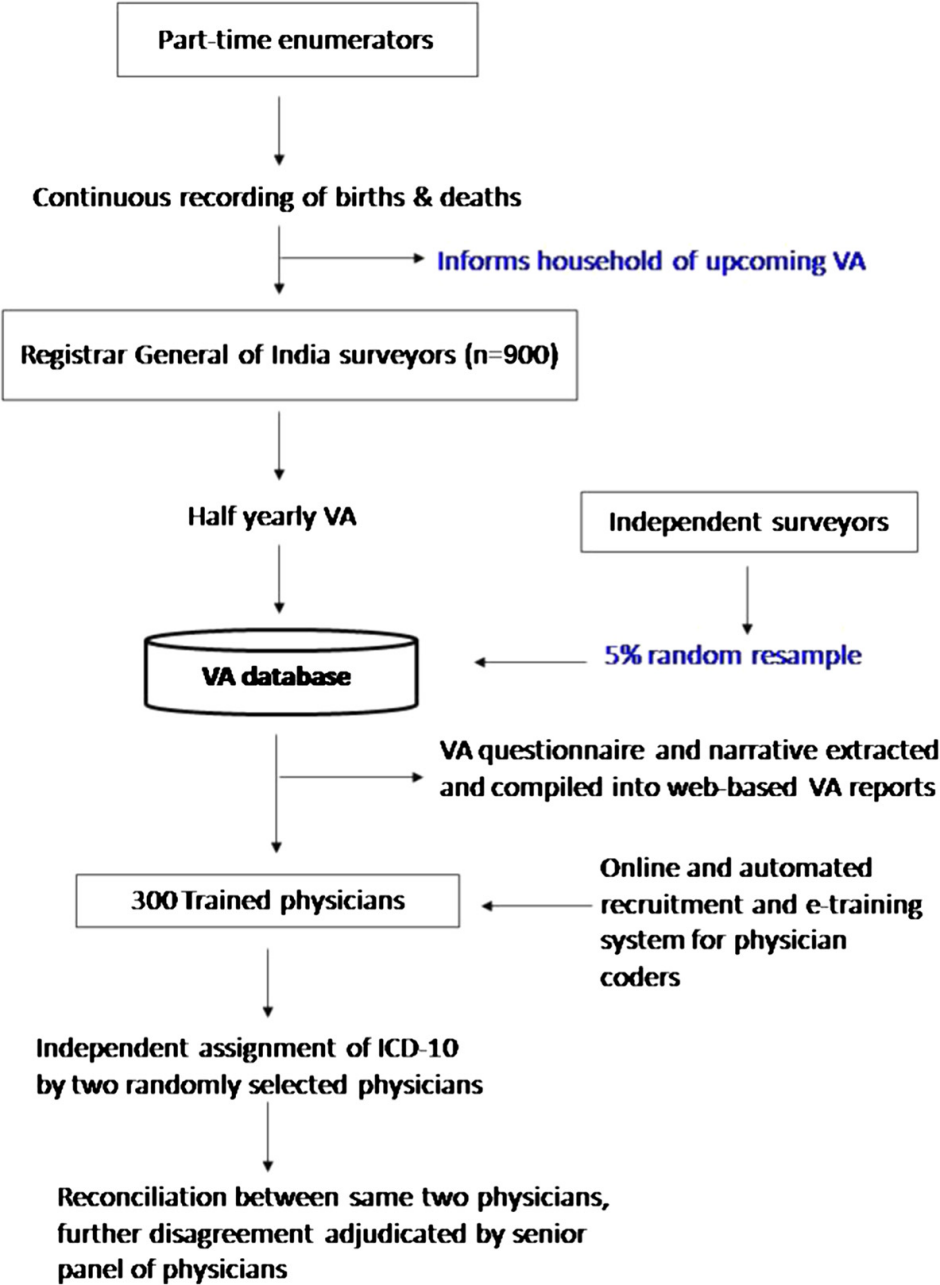
**MDS flow of activities.** To date, about 700,000 deaths have been surveyed and 200,000 deaths have been double coded. The eventual numbers covered will include about 350,000 deaths from 1997 to 2003, of which half will have used the RHIME instrument, and about 650,000 deaths from 2004 to 2014. MDS, Million Death Study; RHIME, routine, reliable, representative, resampled household investigation of mortality with medical evaluation.

The RGI divides India into one million small areas of about 1,000 people on the basis of the decennial national census. The SRS randomly selects about 8,000 of these small areas and monitors all births and deaths in about 1.3 million homes by local, part-time enumerators [7]. Every six months one of about 900 non-medical RGI surveyors visits the homes in which a death had been recorded (Figure 1) and obtains information about the death as well as marital status, occupation, education, alcohol use, and other risk factors [9]. The underlying cause of each death is sought by using an enhanced form of VA, known as the routine, reliable, representative, resampled household investigation of mortality with medical evaluation (RHIME) [6,9]. The RHIME method is a structured investigation of events before death, including a written report in the local language of the household, with various quality controls. The RHIME method relies on the assumption that most CODs have distinct symptoms and signs that can be recognized, recalled and reported by household members or associates of the deceased to a trained, non-medical field worker. Each two-page written report is converted into an electronic record and assigned randomly to 2 of 300 specially trained physicians (stratified only by their ability to code in the local language of the narrative) who independently and anonymously assign an ICD-10 code for the underlying COD using clinical guidelines [23]. If the two physicians initially disagree, they are required to anonymously reconcile by exchanging ICD-10 codes and keywords. Any remaining disagreements are sent to a third, senior physician who adjudicates. About 3% to 5% of the fieldwork is randomly resampled by an independent team and coded in the same way as the main MDS records.

### Key features of the MDS

Table 1 describes the key MDS features designed to increase quality and efficiency of the final COD results. The most important study feature is the use of a true random sample of deaths in India using the SRS sampling framework (which uses continuous enumeration of households with no replacement). The key features of field work include: electronic training of field staff on proper field procedures; use of structured questions plus a half-page narrative in local language (Additional file 1), suitable for physician coding [24,25]; and random resampling of the field work to ensure field staff adhere to proper surveying methods.

**Table 1:** Key features of the Million Death Study

**Feature**	**Purpose**
DESIGN	
Random sample of deaths surveyed	Ensures results are representative of India (based on rural and urban strata for major states, and at the state level for smaller states)
Continuous enumeration of deaths and births	Ensures follow-up of the same houses to enable prospective analyses of risk factors (such as education, smoking and alcohol), and familiarity by households to the SRS field staff
FIELD PROCEDURES	
3% to 5% random household resample of deaths by independent team	Quality check on the reliability of data, and is a disincentive for faulty field work
Structured survey questions, half-page local language narrative, and guiding cardinal symptom lists	Guides surveyors to fully capture chronology of key symptoms by age group, so as to aid physician diagnosis
Extraction of VA field data into web-based reports for coding	Concise reports increase speed and efficiency of coding, custom extraction of data retains confidentiality
PHYSICIAN CODING PROCEDURES	
Independent, anonymous and random physician double coding (stratified only by language)	Increases cross-state comparability (in particular for about half the records which are recorded in Hindi or English), and decreases local biases in coding
Web-based centralized medical coding application, with logical checks, clinical guidelines, and differential diagnoses	Coding application with a user interface which includes searchable ICD-10 codes, standardised clinical guidelines and differential diagnoses, age/sex restrictions (for example, no cervical cancer in males, or senility before old age), and highlighting of keywords; increases the speed, repeatability, and quality of coding versus a paper-based system
Reconciliation and adjudication stages for coding disagreements	Double coding with reconciliation and adjudication helps train new coders on correct use of coding, is a check on coding quality, and a disincentive for faulty coding
Financial incentives for quality of coding	Payment is made per record that has cleared the reconciliation stage rather than per code assigned, thus decreasing incentives for random or faulty coding
Online recruitment and e-training for physicians (http://www.cghr.org/index.php/training/training-centre/)	Physicians train remotely as their schedule allows and are evaluated before entering the system; increases efficiency and quality of coding

The key features of physician coding include: a fully electronic process for physician recruitment, training, evaluation, and certification (Additional file 2); random assignment of records to physicians sorted only by language; customized, open-source software with a helpful user interface for physicians to automatically retrieve and code records, including coding guidelines based on expert criteria [23], and suggested differential diagnoses for major CODs (the top three alternative diagnoses, based on analysis of earlier MDS physician disagreements in over 120,000 deaths); anonymous double-coding of all VA records, with disagreements subject to reconciliation (where each physician sees the key words and diagnosis of the other physician, and can retain or revise her/his ICD-10 assignment); anonymous adjudication of any remaining disagreements (where the adjudicator sees the logical process of key words and diagnoses assigned in the previous coding rounds); unrestricted use of more than 2,000 3-digit ICD-10 codes, including a search function to find any specific code; and age and gender checks for incorrect coding (such as cervical cancer in males).

### Statistical analyses

We analyzed differences in cause-specific mortality fractions (CSMFs) for 19 major COD groups (18 groups based on the three-character ICD-10 codes for families A-Y, and a group for ill-defined causes, comprised mainly of R codes representing ‘symptoms, signs and abnormal clinical and laboratory findings, not elsewhere classified’). Additional files 3 and 4 show how the MDS classification system collapses the individual ICD-10 codes into these 19 causes, which is similar to the system used by the World Health Organization (WHO) [26]. The 2010 version of the Global Burden of Disease (GBD) project [27] uses a more complex procedure that, among other features, utilizes three and four character ICD-10 codes and reassigns ill-defined codes into non ill-defined categories. CSMF is simply the proportion of a given cause out of all deaths and is a useful indicator to compare the population distributions of CODs across various samples [28]. We calculated odds ratios to compare the CSMFs for each of the 19 causes in resampled versus original MDS deaths, hospital- versus home-based deaths, and urban versus rural deaths (with the reference category being the latter for each, respectively). Logistic regression was used to adjust the odds ratios (OR) for age (linear year), sex, religion (Hindu versus other), education (illiterate versus literate), poorer or richer state, and as relevant, hospital versus home, or rural versus urban status. We focus on young and middle age (5 to 69 years) as these deaths are more avoidable [1,29], and thus are of greater public health importance than deaths at older ages (70 years or older). Deaths at 5 to 69 years constituted about 55% of all deaths in India in 2012, according to the United Nations [30]. Details of COD distributions for children under five years of age in the MDS have already been described by age group, sex, and region [12].

## Results

Many factors can influence the validity and reliability of any particular VA system, including the underlying distribution of cause-specific mortality in a given population, the data collection procedures (recall period, interviewer’s characteristics, respondent’s characteristics), and the methods of COD assignment (diagnostic procedures for COD assignment and COD classification system) [28,31]. We provide simple and comprehensive metrics to measure the overall population-level performance of a VA system.

### Ill-defined deaths

The MDS deliberately reports ill-defined CODs, most of which are the ‘R’ codes in the ICD-10. Ill-defined codes are an important indicator of the quality of fieldwork and enable assessment of changes in the quality of field collection and coding over time. Unlike the GBD [27], the MDS does not mix well-defined CODs with re-classified, ill-defined cases. The introduction of the RHIME instrument in 2001 substantially reduced the proportion of ill-defined deaths at ages 5 to 69 in the SRS (where the COD was earlier captured by simply asking the household opinion), from 13% during 1998 to 2000 to below 4% during 2001 to 2003. Misclassification after age 70 years was substantially higher in the SRS, but also dropped from 62% to 18% between the same periods. After age 15, ill-defined deaths were more common in women than in men (data not shown).

### Measuring the underlying distribution of cause-specific mortality

A total of 1,811 deaths at ages 5 to 69 years were randomly re-interviewed in the MDS by independent teams and the records from the resample and the original MDS sample were eventually matched. The CSMF and rank order at ages 5 to 69 years for the 19 major causes in the resampled versus original MDS deaths were similar (Table 2). The adjusted odds ratios differed significantly from unity (OR = 1) for only 3 of the 19 conditions (although this was limited somewhat by the relatively small sample size of the resampled deaths).

**Table 2:** CSMFs by resampled deaths or main MDS deaths at ages 5 to 69 years

	**MDS**		**Resample**		**Rank order**		**OR: resample versus MDS**^**a **^**(95% CI)**	
**Disease**	**Number**	**%**	**Number**	**%**	**MDS**	**Resample**		
Communicable								
Malaria	2,094	3.3	40	2.2	13	16	0.8	(0.6 to 1.1)
Tuberculosis	5,714	9.0	139	7.7	3	4	0.9	(0.8 to 1.1)
HIV/STI	439	0.7	8	0.4	18	19	0.6	(0.3 to 1.2)
Other infectious diseases^b^	7,005	11.1	234	12.9	2	2	1.3	(1.1 to 1.5)
Maternal conditions	1,053	1.7	14	0.8	17	17	0.5	(0.3 to 0.9)
Nutritional conditions	387	0.6	12	0.7	19	18	1.2	(0.7 to 2.1)
Non communicable								
Cancer	5,511	8.7	152	8.4	4	3	0.9	(0.8 to 1.1)
Ischemic heart disease	7,557	12.0	239	13.2	1	1	1.0	(0.9 to 1.2)
Stroke (cerebrovascular disease)	4,526	7.2	137	7.6	6	5	1.0	(0.9 to 1.2)
Other CVD^c^	1,642	2.6	54	3.0	16	15	1.1	(0.8 to 1.5)
Chronic respiratory disease	5,494	8.7	134	7.4	5	6	0.8	(0.7 to 1)
Liver cirrhosis	2,463	3.9	60	3.3	11	13	0.8	(0.6 to 1.1)
Other digestive diseases	2,248	3.6	84	4.6	12	9	1.5	(1.2 to 1.8)
Renal and other endocrine diseases	2,511	4.0	67	3.7	10	11	0.9	(0.7 to 1.1)
Other chronic diseases	1,722	2.7	64	3.5	15	12	1.3	(1 to 1.6)
Injuries								
Road traffic accidents	1,864	3.0	71	3.9	14	10	1.1	(0.8 to 1.4)
Suicides	2,647	4.2	60	3.3	9	13	0.7	(0.6 to 0.9)
Other injuries	4,497	7.1	134	7.4	7	6	1.0	(0.9 to 1.2)
Ill-defined	3,766	6.0	108	6.0	8	8	1.0	(0.8 to 1.2)
Total	63,140	100	1,811	100				

^a^Adjusted for age, sex, religion (Hindu versus not), education (illiterate versus not), state (poorer nine states versus remaining states), rural/urban, and home/hospital death; ^b^respiratory infections, diarrheal diseases, vaccine-preventable diseases, meningitis, encephalitis, tropical diseases, acute bacterial sepsis and severe infections, and other infectious and parasitic diseases; ^c^rheumatic and hypertensive heart disease, heart failure, and other cardiovascular diseases. CI, confidence interval; CSMFs, cause specific mortality fractions; CVD, cardiovascular disease; MDS, Million Death Study; OR, odds ratio; STI, sexually-transmitted infections.

By contrast, there were sharp differences in CSMFs between hospital- and home-based deaths (Table 3). In comparison to home deaths, hospital deaths were more likely to report higher CSMFs for maternal conditions, heart disease, stroke, other cardiovascular diseases, other digestive diseases, road traffic injuries, and other injuries, and more likely to report lower CSMFs for tuberculosis, HIV/STI, chronic respiratory disease, cancer, nutritional conditions, and ill-defined causes. Overall, the adjusted odds ratio differed significantly from unity for 15 of the 19 conditions.

**Table 3:** CSMFs by place of death at ages 5 to 69 years

		**Home**		**Hospital**		**Rank order**		**OR: hospital versus home**^**a **^**(95% CI)**	
**Disease**	**Number**	**%**	**Number**	**%**	**Home**	**Hospital**			
Communicable								
Malaria	1,618	3.7	326	3.0	11	15	1.0	(0.9 to 1.1)
Tuberculosis	4,741	10.8	636	5.9	3	6	0.6	(0.5 to 0.6)
HIV/STI	359	0.8	50	0.5	17	18	0.3	(0.2 to 0.4)
Other infectious diseases^b^	6,430	14.6	1,153	10.7	1	2	0.8	(0.8 to 0.9)
Maternal conditions	522	1.2	343	3.2	16	14	3.4	(2.9 to 4.0)
Nutritional conditions	322	0.7	44	0.4	18	19	0.7	(0.5 to 0.9)
Non communicable								
Cancer	4,183	9.5	984	9.1	5	3	0.8	(0.8 to 0.9)
Ischemic heart disease	4,964	11.3	1,590	14.8	2	1	1.2	(1.1 to 1.3)
Stroke (cerebrovascular disease)	3,290	7.5	906	8.4	6	4	1.3	(1.2 to 1.4)
Other CVD^c^	1,116	2.5	371	3.4	13	12	1.3	(1.1 to 1.4)
Chronic respiratory disease	4,646	10.6	515	4.8	4	9	0.7	(0.6 to 0.7)
Liver cirrhosis	1,707	3.9	545	5.1	10	8	1.0	(0.9 to 1.1)
Other digestive diseases	1,025	2.3	282	2.6	14	17	1.3	(1.2 to 1.5)
Renal and other endocrine diseases	848	1.9	285	2.6	15	16	1.2	(1.1 to 1.4)
Other chronic diseases	2,156	4.9	569	5.3	8	7	0.9	(0.8 to 1.0)
Injuries								
Road traffic accidents	230	0.5	484	4.5	19	11	6.9	(5.8 to 8.1)
Suicides	1,467	3.3	506	4.7	12	10	0.9	(0.8 to 1.0)
Other injuries	1,779	4.0	820	7.6	9	5	1.8	(1.6 to 2.0)
Ill-defined	2,576	5.9	370	3.4	7	13	0.6	(0.5 to 0.7)
Total	43,979	100	10,779	100				

^a^Adjusted for age, sex, religion (Hindu versus not), education (illiterate versus not), state (poorer nine states versus remaining states), rural/urban; ^b^respiratory infections, diarrheal diseases, vaccine-preventable diseases, meningitis, encephalitis, tropical diseases, acute bacterial sepsis and severe infections, and other infectious and parasitic diseases; ^c^rheumatic and hypertensive heart disease, heart failure, and other cardiovascular diseases. Note that most road traffic injury deaths were classified as ‘other’, meaning death did not happen at home or hospital, but at scene of accident or en route elsewhere. CI, confidence interval; CSMFs, cause specific mortality fractions; CVD, cardiovascular disease; MDS, Million Death Study; OR, odds ratio; STI, sexually-transmitted infections.

The rural versus urban comparison (Table 4) showed smaller differences in CSMFs than for hospital versus home comparisons. In comparison to rural deaths, urban deaths were more likely to report higher CSMFs for heart disease, liver cirrhosis and renal and other endocrine diseases, and more likely to report lower CSMFs for malaria, other infectious diseases, maternal conditions, and chronic respiratory diseases. Overall, the adjusted odds ratio differed significantly from unity for 9 of the 19 conditions.

**Table 4:** CSMFs by rural or urban residence at ages 5 to 69 years

	**Rural**		**Urban**		**Rank order**		**OR: urban versus rural**^**a **^**(95% CI)**	
**Disease**	**Number**	**%**	**Number**	**%**	**Rural**	**Urban**		
Communicable								
Malaria	1,857	3.6	237	2.1	11	16	0.8	(0.7 to 0.9)
Tuberculosis	4,870	9.4	844	7.5	3	5	1.0	(0.9 to 1.1)
HIV/STI	365	0.7	74	0.7	18	18	0.9	(0.7 to 1.1)
Other infectious diseases^b^	7,312	14.1	990	8.8	1	3	0.8	(0.7 to 0.8)
Maternal conditions	947	1.8	104	0.9	16	17	0.6	(0.5 to 0.8)
Nutritional conditions	345	0.7	42	0.4	19	19	0.8	(0.6 to 1.1)
Non communicable								
Cancer	4,312	8.3	1,199	10.6	5	2	1.1	(1.0 to 1.2)
Ischemic heart disease	5,509	10.6	2,048	18.1	2	1	1.4	(1.3 to 1.5)
Stroke (cerebrovascular disease)	3,579	6.9	947	8.4	7	4	1.0	(1.0 to 1.1)
Other CVD^c^	1,270	2.4	372	3.3	14	13	1.1	(1.0 to 1.2)
Chronic resp. disease	4,709	9.1	785	6.9	4	6	0.9	(0.8 to 0.9)
Liver cirrhosis	1,857	3.6	606	5.4	11^T^	8	1.4	(1.3 to 1.6)
Other digestive diseases	1,226	2.4	188	1.7	15	9	0.7	(0.6 to 0.8)
Renal and other endocrine diseases	896	1.7	311	2.8	17	15	1.3	(1.1 to 1.4)
Other chronic diseases	2,423	4.7	603	5.3	9	10	1.1	(1.0 to 1.3)
Injuries								
Road traffic accidents	1,559	3.0	518	4.6	13	11	1.1	(0.9 to 1.3)
Suicides	2,280	4.4	367	3.2	10	14	0.7	(0.6 to 0.8)
Other injuries	3,659	7.1	626	5.5	6	7	0.9	(0.8 to 1.0)
Ill-defined	2,866	5.5	440	3.9	8	12	0.8	(0.7 to 1.0)
Total	51,841	100	11,301	100				

^a^Adjusted for age, sex, religion (Hindu versus not), education (illiterate versus not), state (poorer nine states versus remaining states), and home/hospital death; ^b^respiratory infections, diarrheal diseases, vaccine-preventable diseases, meningitis, encephalitis, tropical diseases, acute bacterial sepsis and severe infections, and other infectious and parasitic diseases; ^c^rheumatic and hypertensive heart disease, heart failure, and other cardiovascular diseases. CI, confidence interval; CSMFs, cause specific mortality fractions; CVD, cardiovascular disease; MDS, Million Death Study; OR, odds ratio; STI, sexually-transmitted infections.

### Age-, sex-, and time-plausibility of MDS results

**Figure 2:**
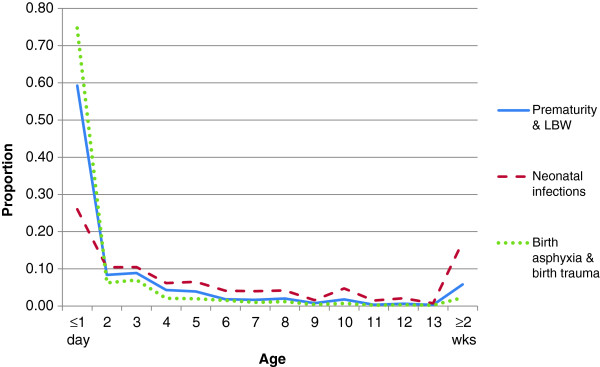
**Proportion of cause-specific neonatal deaths by age (days).** These three conditions account for 80% of all neonatal deaths in India [12].

**Figure 3:**
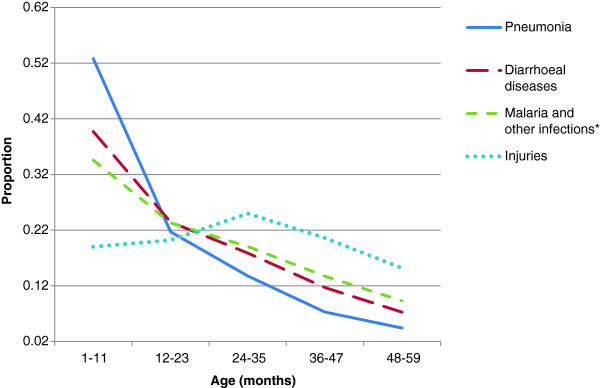
**Proportion of cause-specific child deaths by age (1 to 59 months).** Pneumonia and diarrhea account for 50% of all deaths in India at these ages [12]. *Other infections include sepsis, meningitis, encephalitis, tuberculosis, tetanus, polio, measles, HIV, malaria, other infectious and parasitic diseases, and fever of unknown origin.

**Figure 4:**
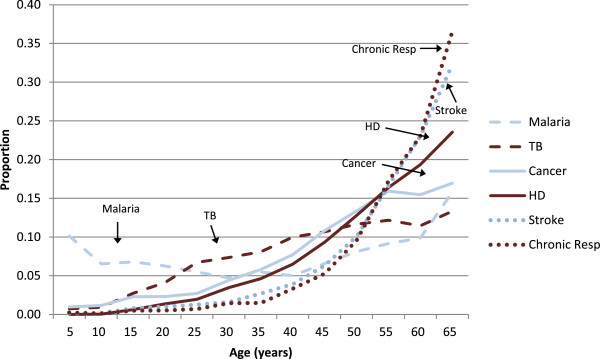
Proportion of selected communicable and non-communicable deaths in adults by age (years).

**Figure 5:**
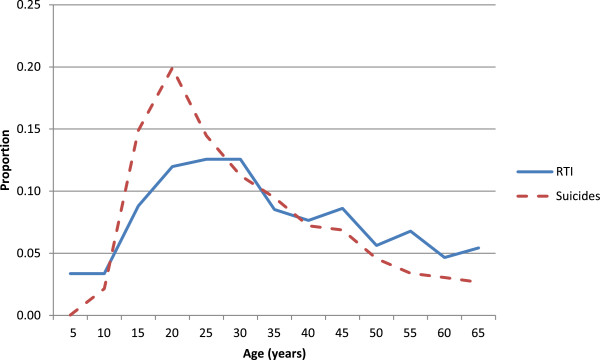
**Proportion of road traffic injury and suicide deaths in adults by age (years).** Road traffic injuries are more common in men than in women [32], but suicide at younger ages is more common in women [16].

**Figure 6:**
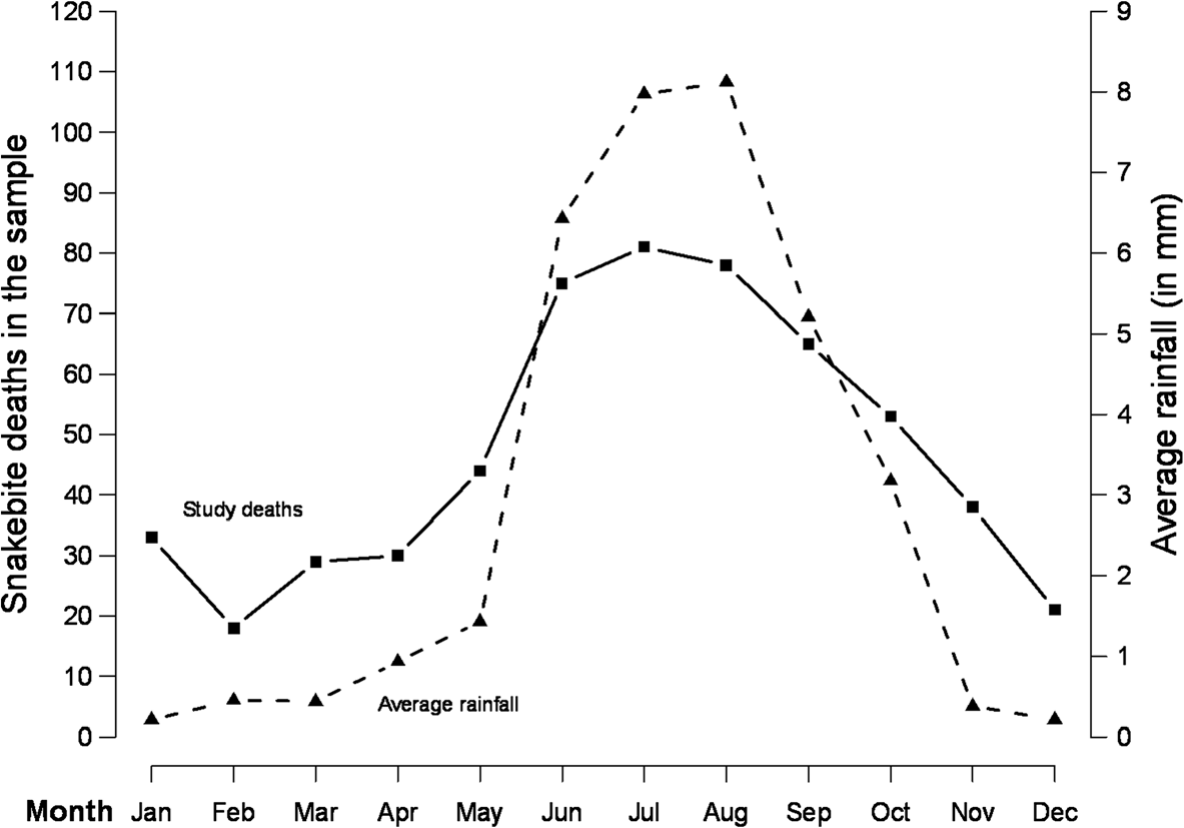
**Seasonality patterns of snakebite mortality and rainfall in states with high-prevalence of snakebite deaths during 2001 to 2003.** Rainfall amount (mm) is cumulative station-wise daily rainfall for the past 24 hours measured at 0830 IST of the day by the India Meteorological Department at its 537 observatories. Maximum and minimum temperatures are also measured daily on the same grid but not presented here. Temporal correlation between snakebite mortality and rainfall: 0.93 (*P* <0.0001), temperature minimum: 0.80 (*P* = 0.002), temperature maximum: 0.35 (*P* = 0.25) [17].

Performance of the MDS or any other VA-based COD system can also be assessed by the age- and sex-plausibility of specific conditions. Details of specific diseases have already been published from the MDS [9-19]. The proportion of cause-specific mortality for each day during the neonatal period (Figure 2) reveals a high proportion of birth asphyxia and birth trauma deaths occurring on the first or second day of life, with neonatal infections occurring in greater proportion during later days of life. Similarly, the proportion of pneumonia deaths is mostly concentrated at ages one to eleven months, with diarrheal diseases less prominent during this time period. Injury deaths in children, particularly among boys, peak at the ages when toddlers become mobile (Figure 3). At ages 5 to 69 years, the age-specific pattern of malaria, tuberculosis, cancer, heart disease and chronic respiratory disease are also plausible (Figure 4), as is the early age of deaths from road traffic injuries, and the peak in early adulthood of suicide (Figure 5). Snakebite deaths closely followed peak rainfall patterns in the high-burden states (Figure 6; [17]).

### Impact of data collection procedures

The MDS showed that the consistency of coding across the broad COD categories or rates of ill-defined causes were not dependent on the household respondents’ level of education or the relationship of the respondent to the deceased (data not shown, [6,33-35]). Similarly, there were no major differences in CSMFs by shorter or longer recall period (deaths occurring within five months to four years earlier; data not shown). The overall CSMF of the top 10 CODs was similar across high- and low-quality narratives (data not shown), emphasizing the point that plausible and replicable population distribution of CODs is possible despite misclassification at the individual level [6,28,31].

### Physician coding patterns in the MDS

Almost two-thirds of deaths at ages 5 to 69 years had immediate agreement on the COD by two independent physicians (Additional file 5a) [33-35]. About half of the remaining physician disagreements were resolved during reconciliation, usually with one physician yielding to the diagnosis of the other. There were no systematic patterns of physician locality, experience, or past coding that predicted which physician yielded (data not shown). The remaining differences at reconciliation were solved by adjudication, usually with the choice of one of the preceding assignments. The proportion requiring adjudication was highest for nutritional conditions, other vascular diseases, other digestive diseases and ill-defined conditions. However, the differences in CSMFs between single coding, double coding or all coding stages combined were small (Additional file 5b).

### Optimal COD classification systems: minimal number of excluded or ill-defined codes

All VA studies group the list of about 12,000 possible ICD-10 codes into a smaller set of key conditions, based on varying criteria (the MDS criteria are shown in Additional file 3). Useful COD classification systems aim to ensure a minimal number of excluded three-character ICD-10 codes in the final tabulation plan and a minimal (but not zero) number of codes classified as ill-defined causes. The WHO VA 2012 standard [26] used a classification system similar to the MDS, while the GBD [27] is more complex. GBD has approximately 156 sub-groups (grouped into 21 larger categories), WHO’s classification system has 63 sub-groups, and the MDS uses 85 sub-groups for CODs above age 5 years, 19 sub-groups for 1 to 59 month deaths and 17 sub-groups for deaths in the first month of life. The MDS child classification conforms mostly to that recommended by the Child Health Epidemiology Reference Group (CHERG) [36]. Table 5 tests these metrics across the MDS, WHO, and GBD systems, by classifying all MDS VA records from 2001 to 2003 (separately for neonates, 1 to 59 months and 5 to 69 years) into these three systems (ages 70 or older were excluded given much higher ill-defined coding rates).

**Table 5:** Completeness of causes of death among three classification systems, using MDS 2001–2003 records

	**Classification system**					
**Causes of death**	**MDS**		**WHO**^**b**^		**GBD**	
	**Number deaths**	**%**	**Number deaths**	**%**	**Number deaths**	**%**
**Neonates (Age 0 to 28 days)**						
Prematurity and low birth weight	3,631	33.3	2,914	26.8	2,604	23.9
Birth asphyxia and birth trauma	2,073	19.0	1,870	17.2	2,064	18.9
Neonatal infections^a^	2,883	26.5	1,261	11.6	1,144	10.5
All other causes	1,600	14.7	4,707	43.2	4,940	45.4
Ill-defined conditions	705	6.5	140	1.3	-	
Deaths missing ICD-10 codes	-	-	-	-	140	1.3
Total deaths: neonates	10,892	100	10,892	100	10,892	100
**Under 5 (Age 1 to 59 month)**						
Pneumonia	3,432	28.0	3,409	27.8	3,410	27.8
Diarrheal diseases	2,716	22.2	2,716	22.2	2,713	22.1
Malaria	587	4.8	587	4.8	587	4.8
Other infections/parasitic diseases	2,149	17.5	2,113	17.2	1,709	13.9
Injuries	757	6.2	757	6.2	722	5.9
All other causes	1,845	15.0	2,280	18.6	1,259	10.3
Ill-defined conditions	774	6.3	398	3.2	-	
Deaths missing ICD-10 codes	-			-	1,860	15.2
Total deaths: 1 to 59 months	12,260	100	12,260	100	12,260	100
**Age 5 to 69 years**						
**Infections, parasitic diseases, maternal and nutritional conditions**						
Malaria	2,094	3.3	2,094	3.3	2,094	3.3
Tuberculosis	5,714	9.0	5,560	8.8	5,713	9.0
HIV/STI	439	0.7	416	0.7	431	0.7
Other infectious diseases	7,005	11.1	7,031	11.1	5,972	9.5
Maternal conditions	1,053	1.7	1,028	1.6	1,028	1.6
Nutritional conditions	387	0.6	391	0.6	381	0.6
**Noncommunicable conditions**		*-*		*-*		*-*
Cancer	5,511	8.7	5,511	8.7	4,048	6.4
Heart Diseases	7,557	12.0	7,231	11.5	7,231	11.5
Stroke	4,526	7.2	4,366	6.9	629	1.0
Other CVD	1,642	2.6	1,723	2.7	471	0.7
Chronic respiratory diseases	5,494	8.7	5,327	8.4	5,367	*8.5*
Cirrhosis of the liver	2,463	3.9	1,705	2.7	1,726	2.7
Other digestive diseases	2,248	3.6	834	1.3	1,285	2.0
Renal/endocrine diseases	2,511	4.0	2,405	3.8	743	1.2
Other chronic diseases	1,722	2.7	6,407	10.1	1,425	2.3
**Injuries**		**-**		**-**		**-**
Road traffic accidents	1,864	3.0	1,990	3.2	1,736	2.7
Suicides	2,647	4.2	2,647	4.2	2,519	4.0
Other injuries	4,497	7.1	4,510	7.1	3,916	6.2
**All other causes**	-	*-*	122	0.2	57	0.1
Ill-defined conditions	3,766	6.0	1,835	2.9	-	-
Deaths missing ICD-10 codes	-	-	7	0.0	16,368	25.9
Total deaths: 5 to 69 years	63,140	100	63,140	100	63,140	100

Notes: We devise a separate classification system in the MDS for neonates aged 0 to 28 days and children aged 1 to 59 months owing to few non-communicable deaths. ^a^Neonatal infections include neonatal pneumonia, sepsis and CNS infections; ^b^in WHO classification, except category 99 (ICD-10 codes R95-R99), all other ill-defined causes and other causes not assigned to major disease categories are pooled with un-specific non-communicable diseases. This table shows only ICD-10, R95-R99 under ill-defined. This table does not show redistributed deaths in the GBD. CVD, cardiovascular disease; GBD, Global Burden of Disease; ICD, International Classification of Disease; MDS, Million Death Study; STI, sexually-transmitted infections; WHO, World Health Organization.

The MDS and WHO systems were able to categorize all the ICD-10 codes assigned for the 10,892 neonatal deaths into broader disease categories, while the GBD system was unable to use the ICD-10 codes from 140 of these deaths. The MDS and WHO systems were able to use the ICD-10 codes from all 12,260 deaths at ages 1 to 59 months, but the GBD system was unable to classify 1,860 (15%) of these deaths. The WHO system was able to capture all but 7 of the 63,140 deaths at ages 5 to 69 years, but the GBD system was unable to capture 16,368 of these (about 25% at these ages). In particular, the GBD system classifies I64 (stroke, not specified as hemorrhage or infarction) as a ‘garbage’ code (meaning a non-useful ICD-10 code which is re-assigned to another cause) and presumably re-classifies this to sub-categories of hemorrhagic or ischemic stroke. Even excluding stroke deaths, the GBD still excluded 12,000 deaths from being classified into a broader disease category. Ill-defined causes (largely those assigned a R code, although a handful of other ICD-10 codes are included) were compared between the three systems. In the MDS, the ill-defined proportions were around 6% for all three age groups. The WHO’s ill-defined rates were the lowest, at 1.3%, 3.2% and 2.9% at ages <1 month, 1 to 59 months, and 5 to 69 years, respectively. By definition, there are zero ill-defined causes in the GBD, as it re-classifies all R-codes to various COD groupings.

The net result is that the MDS classification system assigns a substantially higher CSMF to the grouping of neonatal infections and prematurity in the first month of life than the WHO or GBD classifications (data not shown). At ages 1 to 59 months, the differences between all three systems in CSMF are much smaller. Finally, at ages 5 to 69 years, the three systems yield similar CSMFs for most conditions (leaving aside the anomaly of stroke deaths in the GBD). WHO’s system has a much higher CSMF for other chronic disease deaths, and GBD has a lower CSMF for other infections, cancer, and renal and endocrine diseases (Table 5).

## Discussion

The major global gap in knowledge of causes of death, particularly for adult mortality in LMICs, might be filled by adopting nationally-representative VA surveys [1-5]. We develop and implement simple metrics which can measure the performance of national VA-based COD systems, such as the MDS. Applying these metrics, we find that lay reporting with double physician coding yields plausible results in the MDS. The MDS retains ill-defined deaths as a check on quality, and finds few ill-defined deaths during young and middle age (below age 70 years), but far more at older ages. This corresponds with public health priorities, which are mostly concerned with avoidable death in young and middle age [1,37]. Indeed, misclassification is common at older ages even for medically certified deaths occurring in hospitals in high-income countries [38,39].

A simple but effective method to establish usefulness of CSMFs from VA-based national surveys is to compare if an independent resample yields similar results. The CSMFs and rank order of CODs at the population level is similar between randomly resampled deaths and those from the main MDS. This suggests stability and reproducibility of the RHIME method, but does not itself prove validity. By contrast, CSMFs differed substantially between hospital- and home-based deaths, and between urban and rural deaths, highlighting the need for VA studies to use true random samples to reliably capture COD distribution at the national level. The age-, sex- and temporal-plausibility for major conditions is high. Consistency of coding is not dependent on various household characteristics but does depend on whether the person lived with the deceased and on the availability of a good quality narrative. Physicians reached initial agreement about two-thirds of the time at initial coding. Finally, the MDS classification is roughly comparable to WHO’s classification system in being able to classify most ICD-10 codes assigned to surveyed deaths and to minimize (but not to eliminate) ill-defined conditions, and performs better than the GBD classification system on these metrics.

National patterns of CODs based solely on hospital and/or urban deaths can be misleading. For example, the GBD estimates for India over-report injury deaths, in particular fires, by relying in large part on urban hospital-based deaths [15]. Unpublished data from the MDS also suggests that the leading COD in India (ischemic heart disease) may be overestimated by the GBD given the former’s reliance on urban, hospital deaths. Finally, the MDS shows that the leading cause of cancer death in women in India is cervical, followed by breast. The GBD finds the exact opposite, due to its reliance on mostly urban cancer registries [14]. Hospital-home and urban–rural differences persisted after adjustment for age, education, religion, region and other variables, suggesting there are underlying biases which cannot be easily corrected for, and the need for caution in extrapolating CSMFs from hospitals to non-hospitalized populations. Moreover, there are differences in the distribution of CODs, treatment patterns, and underlying pathogens for infectious causes between hospital deaths (mostly urban) and rural, unattended deaths in the home [1,40-44]. For example, malaria is observed mostly among rural, unattended deaths [11]. Thus, hospital deaths should not be regarded as a gold standard from which to ‘validate’ rural, medically unattended deaths.

VA generally produces a proportion of deaths that are coded as ill-defined or unspecified causes, particularly at older ages. However, ill-defined categories are important to maintain as they permit a check on the quality of a VA system, as well as individual surveyors’ quality of fieldwork [45]. For example, the Indian government ceased an earlier system of obtaining COD from rural health centers [46] in part because the ill-defined rate was rising, suggesting decreasing quality [5,46]. The MDS methods explicitly keep ill-defined codes visible, rather than re-classifying them into other causes and artificially reducing ill-defined codes to zero. The MDS COD classification system groups several of the ill-defined codes with more certain diagnoses (for example, adding R96 for sudden death to the acute myocardial infarction group, see Additional files 3 and 4), though the majority of ill-defined codes remain in a distinct ill-defined group. WHO’s Global Health Estimates (GHE) similarly groups ill-defined codes with other diseases, and the system is reasonably transparent about these re-allocations [47], which are reproducible. The GBD system tends to distribute ill-defined codes to other diseases. GBD uses an unpublished method for re-distributing ill-defined codes to well-defined categories that has not yet been reproduced. 

The simple ICD-10 classification systems used by the MDS and WHO are preferable to the complex re-classification systems used by the GBD. The GBD’s poor performance in classifying deaths is due in part to the peculiar decision to treat cerebrovascular diseases (ICD-10 code I64), as a ‘garbage code’ subject to misclassification. In most VA studies (such as by the INDEPTH network [48,49]), and indeed in the United States [50], I64 constitutes the majority of the cerebrovascular ICD-10 codes (I60-I69) on death certificates.

These findings carry implications for other LMICs considering introduction of VA-based methods. First, simple but important statistical features are to ensure random sampling of deaths, random resampling of fieldwork, and double coding by physicians. The most important limitation in global estimation of CODs is simply an insufficient number of countries that implement simple, large-scale VA studies [51]. The debate about physician versus machine coding is somewhat misleading [52], as it misses the key point that far more nationally-representative studies are needed.

Innovations in electronic capture of field records, as well as electronic physician recruitment, training, certification, and coding, have resulted in the ability to rapidly conduct large physician-coded VA studies. Indeed, the main rate-limiting steps are organizational and financial. Technical innovations can further simplify the fieldwork and ensure physician coding is supported with computer-based diagnosis. The use of electronic data entry [53] with time- and GPS-tracking of fieldwork, as well as the important feature of resampling deaths, can further improve field quality. In the MDS, the major delay in the coding of records has occurred due to administrative issues and reliance on paper-to-electronic scanning, and not due to the rate of physician coding. Advancing the field will also require commitment to open-source materials, methods, and software. To this end, all the MDS tools are freely available to use without restriction. Open-source data sets [54] are the logical next step in the evolution of global estimates of COD.

## Conclusion

Our proposed simple metrics to measure performance of VA-based COD systems are widely applicable in other settings, regardless of whether physician or computer coding is used. However, it would be unrealistic to suggest that MDS-type systems are the only way to improve information on CODs [51]. What matters is the expansion of countries obtaining random samples of COD at national or sub-national levels. The long-term goal remains achievement of full certification of the act of death and medically-certified causes, as is now common in high-income countries [4]. However, in high-income countries, complete coverage took more than 100 years [1,21]. The advent of India’s national unique ID scheme may rapidly accelerate death certification. Over the next few decades, simple VA systems that obtain a random sample of deaths offer an attractive option. Despite the misclassification of VA-based COD systems, they are an order of magnitude better than the current dearth of data on causes of death.

## Competing interests

The authors declare that they have no competing interests.

## Authors’ contributions

All authors contributed equally to this work. LA, WS and PJ conducted the statistical analyses. All authors read and approved the final manuscript.

## Supplementary Material

Additional file 1 **MDS field forms.** Separate two-page forms are used for: (i) neonatal, <28 days; (ii) child, 29 day to 14 years; (iii) adult, 15+ years; and (iv) maternal deaths at ages 15 to 49 years. Click here for file

Additional file 2 **(a) Screenshots from physician e-learning modules, which physicians must complete in their own time before evaluation and eventual certification as a coder in the MDS. (b)** Screenshots from surveyor e-learning modules, which emphasize fieldwork techniques and guidelines to obtain clear and complete information on VA signs and symptoms. Click here for file

Additional file 3 Description of criteria for groupings in the MDS classification system and detailed mappings of ICD-10 codes by subgroup in the MDS, GBD and WHO classification systems. Click here for file

Additional file 4 **Mapping of Indian MDS cause of death categories to GBD 2010 and WHO-VA 2012.** Describes the alignment of cause of death categories that was used for comparison across cause of death classification systems. Click here for file

Additional file 5 **(a) Numbers and proportion of physician agreement (where verbal autopsy records were assigned a final cause of death) by cause of death and stage of coding.** Data based on adult deaths in the MDS. **(b)** Number of records, CSMF, and absolute CSMF error, by cause of death and stage of physician coding. Data based on adult deaths in the MDS. Click here for file
