# Supplementary material for: Performance criteria for verbal autopsy-based systems to estimate national causes of death: development and application to the Indian Million Death Study
Source: BMC Med. 2014 Feb 4;12:21. doi: 10.1186/1741-7015-12-21 (PMC3912490; doi:10.1186/1741-7015-12-21)
Supplement: Additional file 1 — MDS field forms. Separate two-page forms are used for: (i) neonatal, <28 days; (ii) child, 29 day to 14 years; (iii) adult, 15+ years; and (iv) maternal deaths at ages 15 to 49 years. [file 1741-7015-12-21-S1.pdf]

SAMPLE REGISTRATION SYSTEM
SRS - VERBAL AUTOPSY FORM
Form 10A : Neonatal death (28 days or less of age)

CONFIDENTIAL

SRS unit number

Unique form number 1 009958

Year : 20 — 1st HYS — 2nd HYS

Unit name

Name of head of the household — Identification code of the head

Full name of deceased — Identification code of the deceased

Name of mother of the deceased — Identification code of mother of the deceased

Section 1: Details for respondent and deceased

Details of respondent

1. Name of respondent — Identification code of respondent

2. Relationship of respondent with deceased
- 1.
- 2. Brother/Sister
- 3.
- 4. Mother/Father
- 5.
- 6.
- 7.
- 8.
- 9. Grandfather/Grandmother
- 10. Other relative
- 11. Neighbour/No relation

3. Did the respondent live with the deceased during the events that led to death?
1. Yes 2. No

4. Respondent's age in completed years

5. Respondent's sex 1. Male 2. Female

Details of deceased

6. Age in completed days

7. Sex 1. Male 2. Female

8. House address of the deceased (include PIN)

9. Date of death D D / M M / Y Y

10. Place of death?
1. Home 2. Health facility 3. Other place 9. Unknown

11. What did the respondent think this person die of?
(Allow the respondent to tell the illness in his or her own words)

Section 2: Neonatal Death

12A. Did s/he die from an injury or accident? 1. Yes 2. No→*Skip to Q13* 9. Unknown

12B. If yes, what kind of injury or accident?
- 1. Traffic accident
- 2. Falls
- 3. Fall of objects
- 4. Burns
- 5. Drowning
- 6. Poisoning
- 7. Bite/sting
- 8. Natural disaster
- 9. Homicide/assault
- 10. Others

If child died of injury or accident→*Skip to Q41*

Details of pregnancy and delivery

13. Was the child a single or multiple birth?
1. Single 2. Multiple 9. Unknown

14. Where was s/he born?
1. Home 2. Health Facility 3. Others 9. Unknown

15. Who attended the delivery?
- 1. Trained traditional birth attendant
- 2. Untrained traditional birth attendant
- 3. Midwife/Nurse
- 4. Allopathic Doctor
- 5. Ayurvedic/Homeopathic/Unani Doctor
- 6. None
- 7. Other
- 9. Unknown

16. How many months long was the pregnancy?

17A. Was there any complication during the pregnancy, or during labour?
1. Yes 2. No→*Skip to Q18* 9. Unknown

17B. If yes, what complications occurred? (Check all that apply)
- 1. Mother had fits
- 2. Excessive bleeding before/during delivery
- 3. Waters broke one or more days before contractions started
- 4. Prolonged/difficult labour (12 hours or more)
- 5. Operative delivery
- 6. Mother had fever
- 7. Baby delivered bottom or feet first
- 8. Baby had cord around neck
- 9. Unknown

18. Did the mother receive 2 doses of tetanus toxoid during pregnancy?
1. Yes 2. No 9. Unknown

Details of baby after birth

19. Was the baby born alive (alive if the baby ever cried, moved or breathed)?
1. Yes 2. No 9. Unknown

20. Were there any bruises or signs of injury on child's body after the birth?
1. Yes 2. No 9. Unknown

21. Did s/he have any visible malformations at birth (very small head, mass on spine, etc)?
1. Yes 2. No 9. Unknown

22. What was the child's size at birth?
- 1. Very Small
- 2. Smaller than usual
- 3. Average
- 4. Larger than average
- 9. Unknown

23A. Was s/he able to breath immediately after birth?
1. Yes 2. No→*Skip to Q24A* 9. Unknown

23B. If yes, did s/he stop being able to breath/cry?
1. Yes 2. No→*Skip to Q24A* 9. Unknown

23C. If yes, how long (days) after birth did s/he stop breathing/crying?

24A. Was s/he able to suckle normally during the first day of life?
1. Yes 2. No→*Skip to Q25* 9. Unknown

24B. If yes, did s/he stop being able to suck in a normal way?
1. Yes 2. No→*Skip to Q25* 9. Unknown

24C. If yes, how long (days) after birth did s/he stop sucking?

Details of sickness

25. For how many days was s/he sick before death? ☐☐

26A. Did s/he have fever?
☐ 1. Yes ☐ 2. No →*Skip to Q27A* ☐ 9. Unknown

26B. If yes, how many days did the fever last? ☐☐

27A. Did s/he have any difficulty with breathing?
☐ 1. Yes ☐ 2. No →*Skip to Q28A* ☐ 9. Unknown

27B. If yes, for how many days did the difficulty with breathing last? ☐☐

28A. Did s/he have fast breathing?
☐ 1. Yes ☐ 2. No→*Skip to Q29* ☐ 9. Unknown

28B. If yes, for how many days did the fast breathing last? ☐☐

29. Did s/he have in-drawing of the chest?
☐ 1. Yes ☐ 2. No ☐ 9. Unknown

30A. Did s/he have a cough?
☐ 1. Yes ☐ 2. No ☐ 9. Unknown

30B. Did s/he have grunting (demonstrate)?
☐ 1. Yes ☐ 2. No ☐ 9. Unknown

30C. Did his/her nostrils flare with breathing?
☐ 1. Yes ☐ 2. No ☐ 9. Unknown

31A. Did s/he have diarrhoea (frequent liquid stools)?
☐ 1. Yes ☐ 2. No→*Skip to Q32* ☐ 9. Unknown

31B. If yes, for how many days were the stools more frequent or liquid? ☐☐

32. Did s/he vomit?
☐ 1. Yes ☐ 2. No ☐ 9. Unknown

33. Did s/he have redness around, or discharge from, the birth cord stump?
☐ 1. Yes ☐ 2. No ☐ 9. Unknown

34. Did s/he have areas of skin that were red, hot or peeling?
☐ 1. Yes ☐ 2. No ☐ 9. Unknown

35. Did s/he have a skin rash with blisters containing pus?
☐ 1. Yes ☐ 2. No ☐ 9. Unknown

36. Did s/he have yellow eyes or skin?
☐ 1. Yes ☐ 2. No ☐ 9. Unknown

37. Did s/he have spasms or fits (convulsions)?
☐ 1. Yes ☐ 2. No ☐ 9. Unknown

38. Did s/he become unresponsive or unconscious?
☐ 1. Yes ☐ 2. No ☐ 9. Unknown

39. Did s/he have a bulging fontanelle (describe)?
☐ 1. Yes ☐ 2. No ☐ 9. Unknown

40. Did the child's body feel cold when touched?
☐ 1. Yes ☐ 2. No ☐ 9. Unknown

Section 3: Written narrative in local language

41. Narrative language code ☐☐

Please describe the symptoms in order of appearance, doctor consulted or hospitalization, history of similar episodes, enter the results from reports of the investigations, if available.

Respondent's cooperation: ☐ 1. Good ☐ 2. Medium ☐ 3. Poor

Interviewer's name ______ Code ☐☐☐

Date: D D / M M / Y Y

Signature/Impression	
**Respondent**	Respondent
**Interviewer**	Interviewer

SAMPLE REGISTRATION SYSTEM
SRS - VERBAL AUTOPSY FORM
Form 10B : Child death (29 days to 14 years)

CONFIDENTIAL

SRS unit number

Unique form number 2 025980

Year : 20 1st HYS 2nd HYS

Unit name

Name of head of the household

Identification code of the head

Full name of deceased

Identification code of the deceased

Name of mother of the deceased

Identification code of mother of the deceased

Section 1: Details for respondent and deceased

Details of respondent

1. Name of respondent

Identification code of respondent

2. Relationship of respondent with deceased

- 1.
- 2. Brother/Sister
- 3.
- 4. Mother/Father
- 5.
- 6.
- 7.
- 8.
- 9. Grandfather/Grandmother
- 10. Other relative
- 11. Neighbour/No relation

3. Did the respondent live with the deceased during the events that led to death?

1. Yes 2. No

4. Respondent's age in completed years

5. Respondent's sex 1. Male 2. Female

Details of deceased

6. (a) For < 1 year Months

(b) For ≥ 1 year Completed years

7. Sex 1. Male 2. Female

8. Relationship of deceased to head of household

- 1.
- 2. Brother/Sister
- 3. Son/Daughter
- 4
- 5. Grandchild
- 6.
- 7.
- 8.
- 9.
- 10. Other relative
- 11. Neighbour/No relation
- 99. Unknown

9. House address of the decreased (include PIN)

10. Date of death D D / M M / Y Y

11. Place of death?

1. Home 2. Health facility 3. Other place 9. Unknown

12. What did the respondent think this person die of?
(Allow the respondent to tell the illness in his or her own words)

Section 2: Child death

13A. Did s/he die from an injury or accident? 1. Yes 2. No→*Skip to Q14* 9. Unknown

13B. If yes, what kind of injury or accident?

- 1. Traffic accident
- 2. Falls
- 3. Fall of objects
- 4. Burns
- 5. Drowning
- 6. Poisoning
- 7. Bite/sting
- 8. Natural disaster
- 9. Homicide/assault
- 10. Suicide
- 11. Workplace
- 12. Others

If child died of injury or accident → *Skip to Q33A*

Details of baby after birth

14. How was the child's size at birth?

- 1. Very Small
- 2. Smaller than usual
- 3. Average
- 4 Larger than average
- 9. Unknown

15A. Was s/he born premature? 1.Yes 2. No→ *Skip to Q16A* 9. Unknown

15B. If yes, after how many months of pregnancy?

16A. Was the child breast-fed? 1. Yes 2. No→*Skip to Q17* 9. Unknown

16B. If yes, did the child stop feeding during the illness that led to death? 1. Yes 2. No 9. Unknown

Details of sickness

17. How many days was s/he sick before death?

18A. Did s/he have fever? 1.Yes 2. No → *Skip to Q19* 9. Unknown

18B. If yes, how many days did the fever last?

18C. Was the fever accompanied by chills/rigors? 1.Yes 2. No 9. Unknown

19. Did s/he have convulsions or fits? 1.Yes 2. No 9. Unknown

20. Was s/he unconscious during the illness that led to death? 1.Yes 2. No 9. Unknown

21. Did s/he develop stiffness of the whole body? 1.Yes 2. No 9. Unknown

22. Did s/he have a stiff neck? (demonstrate) 1.Yes 2. No 9. Unknown

23A. Did s/he have diarrhoea (more frequent or more liquid stools)? 1. Yes 2. No →*Skip to Q24A* 9. Unknown

23B. If yes, for how many days?

23C. Was there visible blood in the stools? 1. Yes 2. No 9. Unknown

23D. If s/he had diarrhoea, was s/he given any fluids such as (local term for oral rehydration treatment)? 1. Yes 2. No 9. Unknown

24A. Did s/he have a cough? 1. Yes 2. No→*Skip to Q25A* 9. Unknown

24B. If yes, how many days?

24C. Was it...?

1. Dry 2. Productive 3. With blood 9. Unknown

25A. Did s/he have breathing difficulties? 1. Yes 2. No →*Skip to Q26A* 9. Unknown

25B. If yes, for how many days?

25C. Did s/he have fast breathing?
1. Yes 2. No 9. Unknown

25D. Did s/he have in-drawing of chest?
1. Yes 2. No 9. Unknown

25E. Did s/he have wheezing? (demonstrate sound)
1. Yes 2. No 9. Unknown

25F. During the breathing problems, did s/he receive any antibiotics?
1. Yes 2. No 9. Unknown

26A. During the illness, did s/he have abdominal pain?
1. Yes 2. No→***Skip to Q26C*** 9. Unknown

26B. If yes, was the pain in..?
1. All over abdomen 3. Lower
2. Upper 9. Unknown

26C. Did s/he have abdominal distention?
1. Yes 2. No 9. Unknown

27A. Did s/he vomit?
1. Yes 2. No→***Skip to Q28*** 9. Unknown

27B. If yes, for how many days?

28. Did the eye/skin colour change to yellow?
1. Yes 2. No 9. Unknown

29A. Did s/he have any skin disease or rash?
1. Yes 2. No→***Skip to Q30*** 9. Unknown

29B. Was the rash..?
1. All over the body 2. Only on face 9. Unknown

29C. Was this measles (use local term)?
1. Yes 2. No 9. Unknown

30. During the illness that led to death, did s/he become very thin?
1. Yes 2. No 9. Unknown

31. During the weeks preceding death, did s/he suffer from lack of blood or appear pale?
1. Yes 2. No 9. Unknown

32A. Did s/he have repeated illness?
1. Yes 2. No→***Skip to Q33A*** 9. Unknown

32B. If yes, how many illnesses in the past six months?

32C. If yes, what were the common associated symptoms with the illness? (check all that apply)
1. Cough 3. Ear discharge 5. Other
2. Diarrhoea 4. Chills 9 Unknown

33A. Was s/he immunized?
1. Yes 2. No→***Skip to Q34*** 9. Unknown

33B. If yes, did s/he receive BCG injection?
1. Yes 2. No 9. Unknown

33C. If yes, did s/he receive polio drops in the mouth?
1. Yes 2. No 9. Unknown

33D. If yes, did s/he receive an injection for measles (use local term)?
1. Yes 2. No 9. Unknown

Section 3: Written narrative in local language

34. Narrative language code

Please describe the symptoms in order of appearance, doctor consulted or hospitalization, history of similar episodes, enter the results from reports of the investigations, if available.

Respondent's cooperation: 1. Good 2. Medium 3. Poor

Interviewer's name Code

Date: D D / M M / Y Y

Signature/Impression

Respondent Respondent

Interviewer Interviewer

SAMPLE REGISTRATION SYSTEM
SRS - VERBAL AUTOPSY FORM
Form 10C : Adult death (15 years or older)

CONFIDENTIAL

SRS unit number

Unique form number 3 056084

Year : 20 1st HYS 2nd HYS

Unit name

Name of head of the household

Identification code of the head

Full name of deceased

Identification code of the deceased

Section 1: Details for respondent and deceased

Details of respondent

1. Name of respondent

Identification code of respondent

2. Relationship of respondent with deceased

1. Wife/Husband
2. Brother/Sister
3. Son/Daughter
4. Mother/Father
5. Grandchild
6. Son in law/Daughter in law
7. Brother in law/Sister in law
8. Parent in law
9. Grandfather/Grandmother
10. Other relative
11. Neighbour/No relation

3. Did the respondent live with the deceased during the events that led to death?

1. Yes 2. No

4. Respondent's age in completed years

5. Respondent's sex 1. Male 2. Female

Details of deceased

6. Age in years

7. Sex 1. Male 2. Female

8A. For work does s/he have to live away from home?

1. Yes 2. No 9. Unknown

8B. If yes, how many months a year?

1. Less than one month
2. One to three months
3. More than three months

9. Relationship of deceased to head of household

1. Wife/Husband
2. Brother/Sister
3. Son/Daughter
4. Mother/Father
5. Grandchild
6. Son in law/Daughter in law
7. Brother in law/Sister in law
8. Parent in law
9. Grandfather/Grandmother
10. Other relative
11. Neighbour/No relation
12. Self
99. Unknown

10. House address of the deceased (include PIN)

11. How many years did the deceased live at the address?

12. Date of death D D / M M / Y Y

13. Place of death?

1. Home
2. Health facility
3. Other place
9. Unknown

14. What did the respondent think this person die of?
(Allow the respondent to tell the illness in his or her own words)

Section 2: Past History

Had a doctor EVER stated that the deceased had the following diseases?

	Yes	No	Unknown
15. Hypertension			
16. Heart disease			
17. Stroke			
18. Diabetes			
19. Tuberculosis			
20. HIV/AIDS			
21. Cancer (write site in narrative)			
22. Asthma			
23. Other chronic illness (specify in narrative)			

24. During the last year, did the weight of the deceased change significantly?

1. About same
2. Yes, gained significantly (gained 2.5 Kg or more)
3. Yes, lost significantly (lost 2.5 Kg or more)
9. Unknown

25A. Was the deceased taking any medications regularly during the last five years?

1. Yes 2. No 9. Unknown

25B. If yes, write names of upto three medicines. (In Hindi or English only)

First, ask the following questions for the deceased (First column), and then ask them for the main respondent (Second column)

Tobacco, alcohol and diet	Deceased *(Ask first)*	Respondent *(Ask second)*
26A. Did s/he smoke tobacco within the last 5 years?	1. Definite Yes 2. Definite No 9. Unknown	1. Definite Yes 2. Definite No 9. Unknown
26B. If yes, how many bidi per day?		
26C. If yes, how many cigarettes per day?		
26D. Any other tobacco smoked?	1. Definite Yes 2. Definite No 9. Unknown	1. Definite Yes 2. Definite No 9. Unknown
27A. Did s/he chew tobacco within the last 5 years?	1. Definite Yes 2. Definite No 9. Unknown	1. Definite Yes 2. Definite No 9. Unknown
27B. Did s/he apply tobacco within the last 5 years?	1. Definite Yes 2. Definite No 9. Unknown	1. Definite Yes 2. Definite No 9. Unknown
28A. Did s/he normally drink alcohol (use local term) at least once a week during most weeks?	1. Definite Yes 2. Definite No 9. Unknown	1. Definite Yes 2. Definite No 9. Unknown
28B. If yes, normal average no. of days per week drink was taken. (1 to 7, or 9. Unknown)		
29. Was s/he a pure vegetarian (consumed no egg, meat or fish) for last few years?	1. Definite Yes 2. Definite No 9. Unknown	1. Definite Yes 2. Definite No 9. Unknown

For, female deaths aged 15-49 ask the following questions. For all others, skip to Q31

30A. Was she either known or suspected to be pregnant?

1. Yes 2. No

30B. Did she die within 42 days of delivery? 1. Yes 2. No

30C. Did she die within 42 days of abortion? 1. Yes 2. No

If YES to question Q30A, B or C then DO NOT complete narrative below. Instead complete Form 10D and copy the Form 10D number here. → **5**

Section 3: Written narrative in local language

31. Narrative language code

Please describe the symptoms in order of appearance, doctor consulted or hospitalization, history of similar episodes, enter the results from reports of the investigations, if available.

Respondent's cooperation: 1. Good 2. Medium 3. Poor

Interviewer's name Code

Date: D D / M M / Y Y

Signature/Impression

Respondent Respondent

Interviewer Interviewer

SAMPLE REGISTRATION SYSTEM
SRS - VERBAL AUTOPSY FORM
Form 10D : Maternal death (females aged 15 to 49 years)

CONFIDENTIAL

SRS unit number

Copy this number on the Form 10C 5504352

Year : 20   1st HYS   2nd HYS

Unit name

Name of head of the household

Identification code of the head

Full name of deceased

Identification code of the deceased

1A. Was she pregnant?
1. Yes 2. No 9. Unknown

1B. If yes, how many months?

If woman died within 42 days of delivery/abortion

2A. Did she receive antenatal care during the pregnancy?
1. Yes 2. No 9. Unknown

2B. How many times did she receive antenatal care during the pregnancy?

3. How many days before death did she deliver/abortion?

4. Where was the delivery/abortion?
1. Home 3. Other
2. Health facility 9. Unknown

5. Who attended the delivery?
1. Trained traditional birth attendant 5. Ayurvedic/Homeopathic/ Unani Doctor
2. Untrained traditional birth attendant 6. None
3. Midwife/Nurse 7. Other
4. Allopathic Doctor 9. Unknown

If the woman had abortion, skip to Q18

6. Did she have a caesarean delivery?
1. Yes 2. No 9. Unknown

7. Did she have too much bleeding at the beginning of labour pains?
1. Yes 2. No 9. Unknown

8. Did she have too much bleeding during labour (before delivering the baby)?
1. Yes 2. No 9. Unknown

9. Did she have too much bleeding after delivering the baby?
1. Yes 2. No 9. Unknown

10. Did she have prolonged labour > 12 hrs?
1. Yes 2. No 9. Unknown

11. Did she have difficulty in delivering the baby?
1. Yes 2. No 9. Unknown

12. Did she have a forceps or vacuum delivery?
1. Yes 2. No 9. Unknown

13. Did she have difficulty in delivering the placenta?
1. Yes 2. No 9. Unknown

14. Did she have fits and loss of consciousness?
1. Yes 2. No 9. Unknown

15. Did she have fits during pregnancy/during labour or after labour?
1. Yes 2. No 9. Unknown

16. Did she have fever?
1. Yes 2. No 9. Unknown

17. Did she have foul smelling discharge?
1. Yes 2. No 9. Unknown

Section 3: Written narrative in local language

18. Narrative language code

Please describe the symptoms in order of appearance, doctor consulted or hospitalization, history of similar episodes, enter the results from reports of the investigations, if available.

Respondent's cooperation: 1. Good 2. Medium 3. Poor

Interviewer's name   Code

Date: D D / M M / Y Y

Signature/Impression

Respondent   Respondent

Interviewer   Interviewer
