# Supplementary material for: Performance criteria for verbal autopsy-based systems to estimate national causes of death: development and application to the Indian Million Death Study
Source: BMC Med. 2014 Feb 4;12:21. doi: 10.1186/1741-7015-12-21 (PMC3912490; doi:10.1186/1741-7015-12-21)

# Additional file 2a – Screenshots of physician training e-learning modules

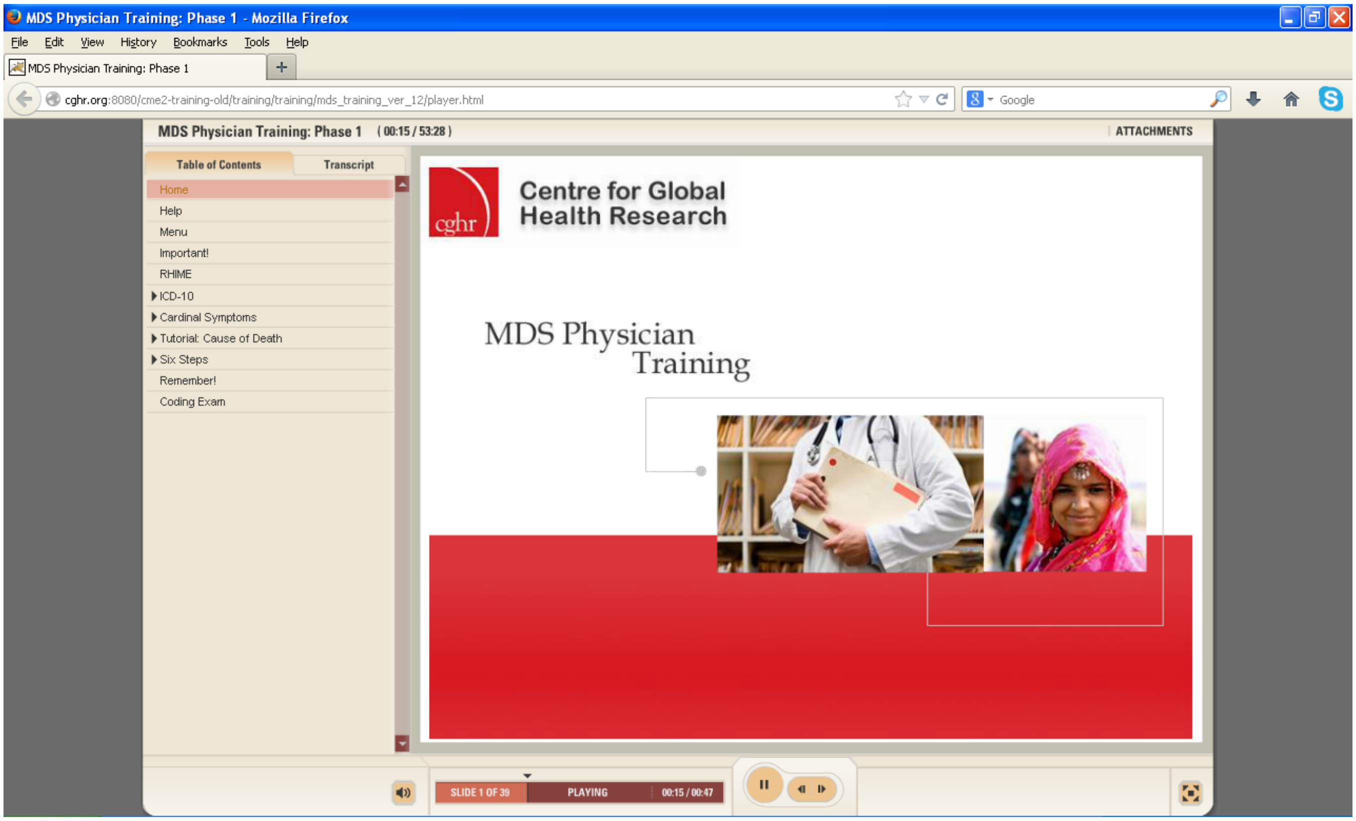


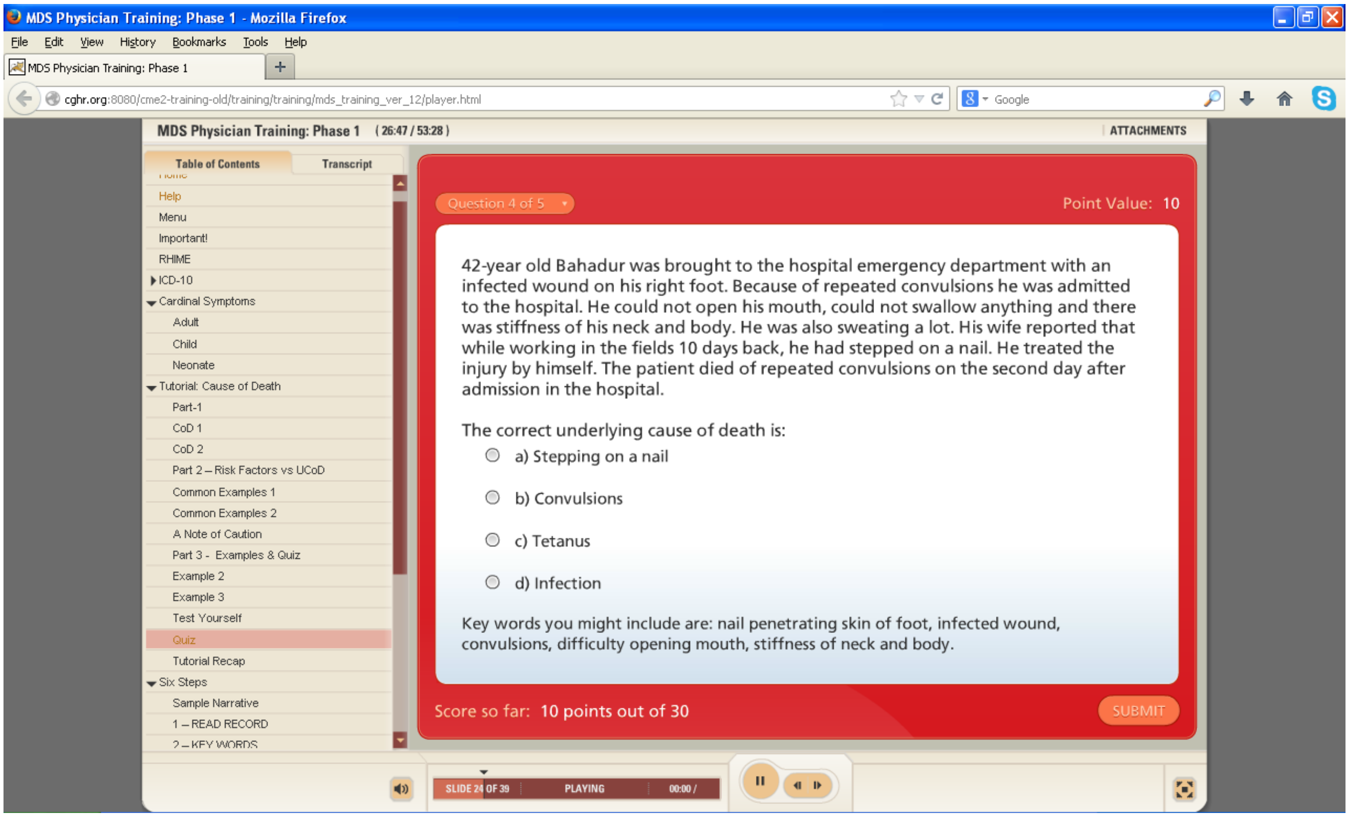

# Additional file 2b – Screenshots of verbal autopsy surveyors' e-learning modules

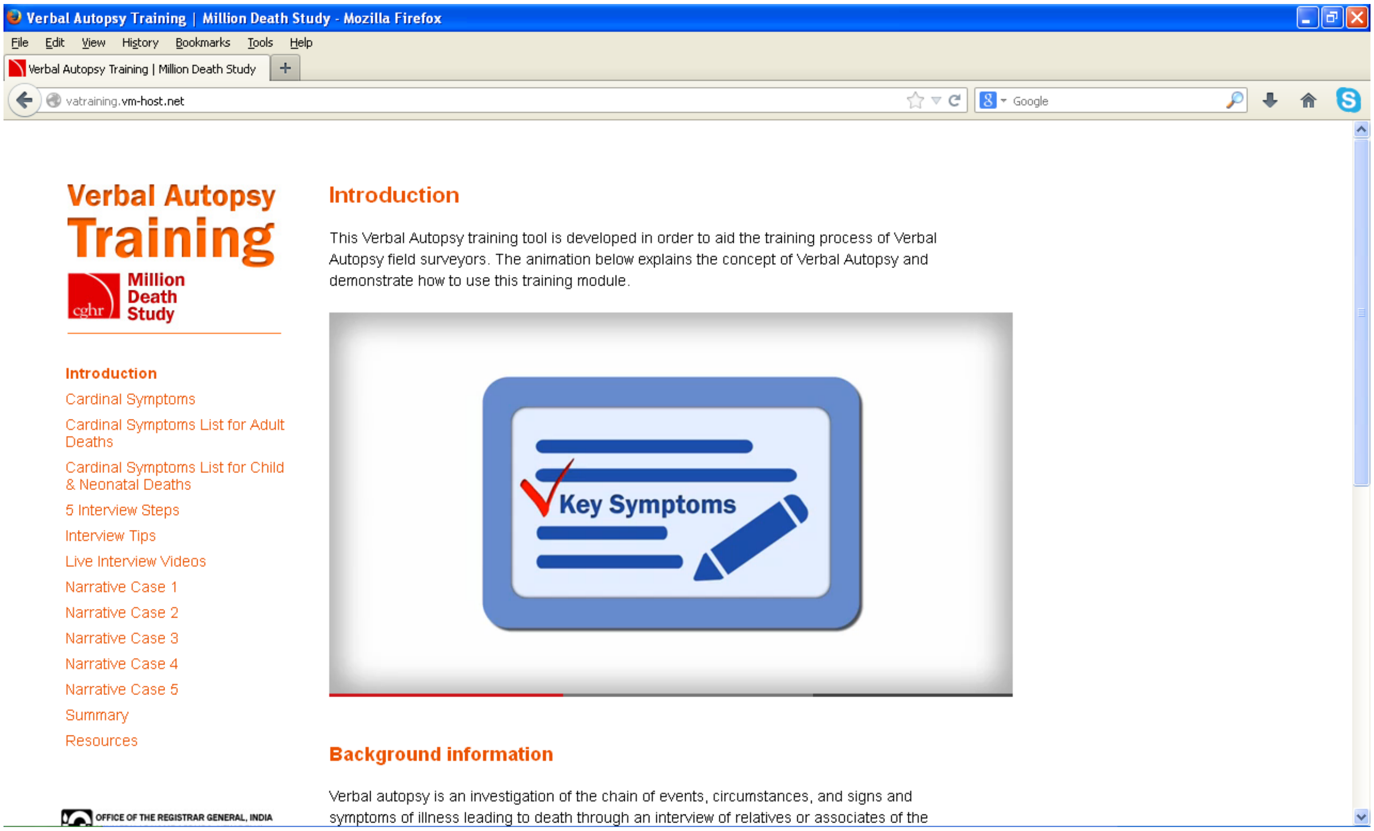


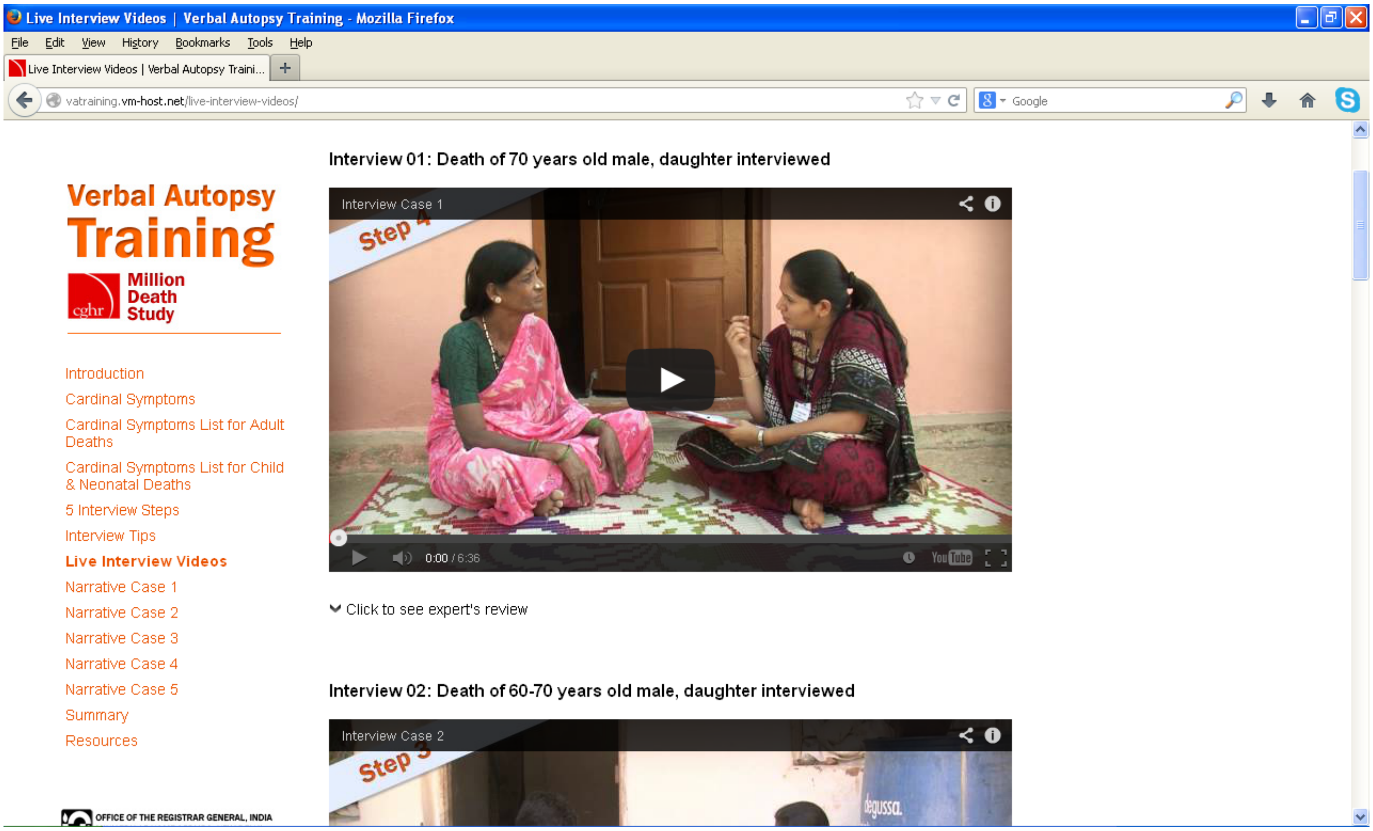

Supplement: Additional file 2 — (a) Screenshots from physician e-learning modules, which physicians must complete in their own time before evaluation and eventual certification as a coder in the MDS. (b) Screenshots from surveyor e-learning modules, which emphasize fieldwork techniques and guidelines to obtain clear and complete information on VA signs and symptoms. [file 1741-7015-12-21-S2.pdf]
