# Supplementary material for: Performance criteria for verbal autopsy-based systems to estimate national causes of death: development and application to the Indian Million Death Study
Source: BMC Med. 2014 Feb 4;12:21. doi: 10.1186/1741-7015-12-21 (PMC3912490; doi:10.1186/1741-7015-12-21)
Supplement: Additional file 3 — Description of criteria for groupings in the MDS classification system and detailed mappings of ICD-10 codes by subgroup in the MDS, GBD and WHO classification systems. [file 1741-7015-12-21-S3.pdf]

Additional File 3: Description of criteria for groupings in the MDS classification system

The ICD-10 has over 16,000 codes available, and it's replacement, ICD-11 will have a comparable number. These long lists are not suitable for verbal autopsy, where causes of death cannot be ascertained in this great of detail. Using an expert panel, we focused on combining the main-level 3-digit ICD codes into 85 broader categories using the following guidelines: those presenting overlapping signs and symptoms (i.e., meningitis and encephalitis, and prematurity and low birth weight); shared programmatic implications for disease management (i.e., vaccine preventable diseases); those where a common organ, physiological system or pathological process was involved (i.e., cardiovascular disease, cancer by site or point of origin), including non-specific R codes pertaining to an organ system; those commonly found in particular gender and age groups (i.e., maternal and perinatal causes); and finally, ill-defined diseases where a specific organ system could not be identified, or cases of senility and unclassified death. These 85 categories were further grouped into 18 of the major conditions, plus ill-defined deaths. Additional Table 2 shows the detailed ICD-10 codes used in the MDS classification system.

MDS adult cause of death classification (ages 15+ years)

Codex4	Codex2	Codex	Disease	ICD 10 range
				A00-A99,B00-B17,B19-B99,C46,D50-D53,D64,D84,E00-E02,E40-E64,F53,G00-G09,H10,H60,H65-H68,H70-H71,I30,I32-I33,I39-I41,J00-J22,J32,J36,J65,J85-J86,K02,K04-K05,K61,K65,K67,K81,L00-L04,L08,M00-M01,M60,M86,N10,N30,N34,N41,N49,N61,N70-N74,O00-O99,P00-P96,R04,R50,R75,R95-R96,U00,U04,X53-X54,Y95
1			Communicable, maternal, perinatal and nutritional conditions	
	1A		**Tuberculosis**	**A15-A19, J65, B90**
		1A01	Tuberculosis	A15-A19, B90,J65
	1B		**HIV/AIDS**	**B20-B24,C46,D84 R75**
		1B01	HIV/AIDS	B20-B24,C46,D84 R75
	1C		**Sexually-transmitted infections excl. HIV/AIDS**	**A51-A64, N70-N74**
		1C01	Syphilis	A51-A53
		1C02	Other sexually transmitted infections (excl. HIV/AIDS)	A54-A64, N70-N74
	1D		**Diarrhoeal diseases**	**A00-A09**
		1D01	Diarrhoeal diseases	A00-A09
	1E		**Selected vaccine preventable diseases**	**A33,A35-A37,A80,B01,B03,B05-B06,B26,B91**
		1E01	Tetanus	A33,A35
		1E02	Measles	B01,B05
		1E03	Poliomyelitis	A80, B91
		1E04	Other vaccine preventable diseases	A36-A37,B03,B06,B26
	1F		**Meningitis/encephalitis**	**A39,A81-A89,G00-G09**
		1F01	Meningitis/encephalitis	A39, A81,A83-A89, G00-G09
		1F02	Rabies	A82
	1G		**Hepatitis**	**B15-B17, B19**
		1G01	Hepatitis	B15-B17,B19
	1H		**Malaria**	**B50-B54**
		1H01	Malaria	B50-B54
	1I		**Selected tropical diseases**	**A30, A71,A90-A99,B55-B58, B60-B83,B92**
		1I01	Protozoal diseases	B55-B58, B60-B64
		1I02	Leprosy	A30, B92
		1I03	Arthropod-borne viral fevers	A90-A99
		1I04	Trachoma	A71
		1I05	Helminthiases	B65-B83
	1J		**Respiratory infections**	**H65-H68,H70-H71,J00-J22,J32,J36,J85-J86,P23,U04**
		1J01	Acute respiratory infections	H65-H68,H70-H71,J00-J22,J32,J36,J85-J86,P23,U04
	1K		**Acute bacterial sepsis & severe Infections**	**A20-A28,A32,A38,A40-A49,A68,A70,A74-A79,B95-B96,H10,H60,I30,I32-I33,I39-I41,K02,K04-K05,K61,K65,K67,K81,L00-L04,L08,M00-M01,M60,M86,N10,N30,N34,N41,N49,N61,P36-P39**
		1K01	Severe Systemic Infection	A20-A28,A32,A38,A40-A49,A68,A70,A74-A79,B95-B96,P36-P39,U80-U89
		1K02	Severe Localized Infection	H10,H60,I30,I32-I33,I39-I41,K02,K04-K05,K61,K65,K67,K81,L00-L04,L08,M00-M01,M60,M86,N10,N30,N34,N41,N49,N61
	1L		**Other infectious and parasitic diseases**	**A31,A50,A65-A67,A69,B00, B02,B04,B07-B09,B25,B27-B49,B59,B85-B89,B94,B97-B99,P35,U00,Y95**
		1L01	Other infectious diseases	A31,A50,A65-A67,A69,B00,B02,B04,B07-B09,B25,B27-B49,B59,B85-B89,B94,B97-B99,P35,U00,Y95
	1M		**Maternal conditions**	**A34,O00-O99,A34,F53**
		1M01	Abortion or miscarrage	O00-O08
		1M02	Hypertensive disorders of pregnancy	O11-O16
		1M03	Obastetric haemorrhage	O20,O43-O46,O67,O71-O72
		1M04	Maternal sepsis	O23,O41,O85-O86,O91,A34
		1M05	Other maternal conditions	O10, O21-O22,O24-O40,O42,O47-O66,O68-O70,O73-O84,O87-O90,O92-O99,F53
	1N		**Perinatal conditions**	**P00-P22, P24-P29,P50-P96,R95,R96****
		1N01	Low birth weight/preterm	P01,P05-P07,P22,P25-P28,P52,P61,P77
		1N02	Birth asphyxia and birth trauma	P02-P03, P10-P15, P20-P21,P24,P50,P90-P91
		1N03	Other perinatal conditions	P00, P04, P08,P29, P51,P53-P60,P70-P76,P78-P83,P92-P94, P96,R95,R96** (** includes sudden infant death : R96 < 1 year)
	1O		**Nutritional deficiencies**	**D50-D53,D64,E00-E02, E40-E46,E50-E64,X53-X54**
		1O01	Protein-energy malnutrition	E40-E46,X53-X54
		1O02	Iron, vitamin deficienies and nutritional anaemias	D50-D53,D64,E00-E02,E50-E64
	1P		**Fever of unknown origin**	**R50**
		1P01	Fever of unknown origin	R50

B18,C00-C45,C47-C97,D00-D48,D55-D63,D65-D83,D86-D89,E03-E35,E65-E90,F00-F52,F54-F99,G10-G99,H00-H06,H11-H59,H61-H62,H69,H72-H95,I00-I28,I31,I34-I38,I42-I99,J30-J31,J33-J35,J37-J64,J66-J84,J90-J99,K00-K01,K03,K06-K60,K62-K63,K66,K70-K80,K82-K93,L05,L10-L99,M02-M54,M61-M85,M87-M99,N00-N08,N11-N29,N31-N33,N35-N40,N42-N48,N50-N60,N62-N64,N75-N99,Q00-Q99,R00-R01,R03,R05-R06,R10-R23,R26-R49,R55-R56,R59,R63,R70-R74,R76-R77,R80-R82,R84-R87,R90-R91,R96**,X45,Y15,Y90-Y91

2		Noncommunicable diseases		
2A		Neoplasms	C00-C45,C47-C97,D00-D48,N60,N62-N64,N87,R59	
	2A01	Upper aerodigestive		C00-C15,C30-C32,D10-D11
	2A02	Stomach		C16
	2A03	Colon, rectum and small intestine		C17-C21,D12
	2A04	Liver		C22
	2A05	Other digestive		C23-C26,C48,D00-D01,D13,D37
	2A06	Lung & airway cancers		C33-C34,C39,D02,D14
	2A07	Breast cancer		C50,D05,D24,N60, N62-N64
	2A08	Cervix & Uterus cancers		C53-C55,D06,N87
	2A09	Other female genital cancer		C51-C52,C56-C58,D25-D28,D39
	2A10	Male genital cancers		C60-C63,D29,D40
	2A11	Urinary		C64-C68, D30,D41
	2A12	Brain and eye		C69-C72,D31-D33,D42-D43
	2A13	Leukemias, lymphomas and oth. hemotopoitic malignancie		C81-C96,D45-D47
	2A14	Other neoplasms		C37-C38,C40-45,C47,C49,C73-C75,C77-C79,C97,D03-D04,D07-D09,D15-D23,D34-D36,D38,D44,R59
	2A15	Cancer with unspecified sites		C76,C80,D48
2B		Diabetes mellitus	E10-E14	
	2B01	Diabetes mellitus		E10-E14
2C		Endocrine and immune disorders	D55-D63,D65-D83,D86-D89,E03-E07,E15-E16,E20-E35,E65-E90,R70-R74,R76-R77,R81	
	2C01	Endocrine and immune disorders		D55-D63, D65-D83, D86-D89, E03-E07, E15-E16, E20-E35, E65-E90,R70-R74,R76-R77,R81
2D		Neuro-psychiatric conditions	F00-F09,F11-F52,F54-F99, G10-G44,G47-G80,G90-G99,R26-R29,R40-R49,R56,R90	
	2D01	Epilepsy		G40-G41,R56
	2D02	Other neuropsychiatric disorders		F00-F09,F11-F39,F40-F52,F54-F99,G10-G37,G43-G44,G47-G73, G80,G90-G99,R26-R29,R40-R49,R90
2F		Skin, Musculoskeletal, Sensse Organ, Oral disorders	H00-H06,H11-H59,H61-H62,H69,H72-H95,K00-K01,K03,K06-K14,L05,L10-L99,M02-M54,M61-M85,M87-M99,R20-R23	
	2F01	Skin diseases		L05,L10-L99,R20-R23
	2F02	Musculoskeletal disorders		M02-M54,M61-M85,M87-M99
	2F03	Sense organ disorders		H00-H06,H11-H59,H61-H62,H69,H72-H95
	2F04	Oral conditions		K00-K01,K03,K06-K14
2G		Cardiovascular diseases	G45-G46,G81-G83,I00-I28,I31,I34-I84,I86-I99,R00-R01,R03,R55,R96**	
	2G01	Rheumatic heart disease		I00-I09,I38
	2G02	Hypertensive heart diseases		I10-I15
	2G03	Ischaemic heart diseases		I20-I25,I44,I46,I70,R55,R96**
	2G04	Cerebrovascular disease		I60-I69,G45-G46,G81-G83
	2G05	Heart failure		I50
	2G06	Other cardiovascular diseases		I26-I28,I31,I34-I37,I42-I43,I45,I47-I49,I51-I52,I71-I84,I86-I99,R00-R01,R03
2H		Chronic Respiratory diseases	J30-J31,J33-J35,J37-J64,J66-J84,J90-J99,R04-R06,R84,R91	
	2H01	Asthma and Chronic obstructive pulmonary disease		J40-J47,J63,J93
	2H02	Other chronic respiratory diseases		J30-J31,J33-J35,J37-J39,J60-J62,J64,J66-J84,J90-J92,J94-J99,R04-R06,R84,R91
2J		Digestive diseases	B18,F10,I85,K20-K60,K62-K63,K66,K70-K80,K82-K93,R10-R19,R63,R85,X45,Y15,Y90,Y91	
	2J01	Gastro-oesophageal		K20-K23,K25-K31,I85,R12,R13
	2J02	Lliver and alcohol related diseases		B18,F10,K70-K77,R16-R18,X45,Y15,Y90-Y91
	2J03	Other digestive diseases		K35-K38, K40-K60,K62-K63,K66,K80,K82-K93,R10-R11,R14-R15,R19,R63,R85
2K		Genito-urinary diseases	N00-N08,N11-N29,N31-N33,N35-N40,N42-N48,N50-N51,N75-N86,N88-N99,R30-R39,R80,R82,R86,R87	
	2K01	Nephritis and nephrosis		N00-N08,N11-N19
	2K02	Other genitourinary system diseases		N20-N29,N31-N33,N35-N40,N42-N48,N50-N51, N75-N86,N88-N99,R30-R39,R80,R82,R86,R87
2L		Congenital anomalies	Q00-Q99	
	2L01	Congenital anomalies		Q00-Q99

**3**		**Injuries**	**V01-V99,W00-W99,X00-X44,X46-X52,X57-Y14,Y16-Y36,Y40-Y89,Y96-Y98**
**3A**		**Unintentional injuries**	**V01-V99,W00-W99,X00-X44,X46-X52, X57-X59,Y40-Y86,Y88-Y89**
	3A01	Road trafic accidents	V01-V04,V06-V80, V82--V83,V86-V89,V99,Y85
	3A02	Other transport accidents	V05,V81,V84-V85,V90-V98
	3A03	Poisonings	X40-X44,X46-X49
	3A04	Falls	W00-W19
	3A05	Fires	X00-X09
	3A06	Drownings	W65-W74
	3A07	Venomous snakes, animals and plants	X20-X29,W57,W60
	3A08	Other unintentional injuries	W20-W56,W58-W59,W64,W75-W99, X10-X19,X30-X39,X50-X52,X57-X59,Y40-Y84,Y86,Y88-Y89
**3B**		**Intentional injuries**	**X60-Y09,Y35-Y36,Y87**
	3B01	Self-inflicted injuries (suicide)	X60-X84
	3B02	Interpersonal violence and other intentional injuries	X85-Y09,Y35-Y36,Y87
**3C**		**Undetermined Intent**	**Y10-Y14,Y16-Y34,Y96-Y98**
	3C01	Undetermined Intent	Y10-Y14,Y16-Y34,Y96-Y98
**3D**		**Injury and poisoning -* MDS not implemented this catagc**	**Injury and poisoning -* MDS instruction given Not to coad this catagory**
	3D01	Injury and poisoning -* MDS instruction given Not to code t	S00-T98
**4**		**Symptoms, signs and Ill-defined conditions**	**B18,F10,I85,K20-K60,K62-K63,K66,K70-K80,K82-K93,R11-R19,R63,R85,X45,Y15,Y90,Y91**
**4A**		**Ill defined or cause unknown**	**R02,R07-R09,R25,R51-R54,R57-R58,R60-R62,R64-R69,R78-R79,R83,R89,R92-R94,R96**,R98-R99**
	4A01	Senility	R54
	4A02	Other Ill-defined and abnormal findings	R02,R07-R09,R25,R51-R53,R57,R58,R60-R62,R64-R69,R78-R79,R83,R89,R92-R94,R96**,R98
	4A03	Unspecified deaths	R99
**5**		**Factors influencing health status and contact with health services**	**Z00-Z99**
**5A**		**Factors influencing health status and contact with health services (not used in MDS)**	**Z00-Z99**
	5A01	Factors influencing health status	Z00-Z99

MDS child cause of death classification (ages 1-59 months)

CCodex4	CCodex2	CCodex	Disease	ICD 10 range
1			**Communicable, perinatal and nutritional conditions**	
	1A		**Early childhood infections**	**A20-A28,A32,A37-A44,A46,A48-A49,A68-A70,A74-A75,A77-A79,A81-A89,B95-B96,G00-G09,H10,H60,H65-H68,H70-H71,I30,I32-I33,I39-I41,J00-J22,J32,J36,J85-J86,K65,K67,K81,L00-L04,L08,M00-M01,M60,M86,N10,N30,N34,N41,N49,N61,P23,P36,P38,U04**
		1A01	Pneumonia	A37, H65-H68, H70-H71, J00-J22, J32, J36, J85-J86, P23, U04
		1A02	Acute bacterial sepsis and severe infections	A20-A28, A32, A38, A40-A44, A46, A48-A49, A68-A70, A74-A75, A77-A79, B95-B96, H10, H60, I30, I32-I33, I39-I41, K65, K67, K81, L00-L04,L08, M00-M01, M60, M86, N10, N30, N34, N41, N49, N61, P36, P38
		1A03	Meningitis/encephalitis	A39, A81-A89, G00-G09
	1B		**Other communicable diseases**	**A00-A09,A15-A19, A30-A31,A33-A36, A50-A67, A71,A80,A90-A99, B90, J65, B91,B01, B05,B20-B24, R75,B50-B54, B00, B02-B04, B06-B09, B15-B19, B25-B27, B30, B33-B49, B55--B60, B64-B83,B85-B89, B92, B94, B97, B99, K02, K04, K05, K61, N70-N74, P35, P37, P39, U00, Y95R50**
		1B01	Diarrhoeal diseases	A00-A09
		1B02	Tuberculosis	A15-A19, B90, J65
		1B03	Tetanus	A33-A35
		1B04	Poliomyelitis	A80, B91
		1B05	Measles	B01, B05
		1B06	HIV/AIDS	B20-B24, R75
		1B07	Malaria	B50-B54
		1B08	Other infectious and parasitic diseases	A30-A31, A36, A50-A67, A71, A90-A99, B00, B02-B04, B06-B09, B15-B19, B25-B27, B30, B33-B49, B55--B60, B64-B83,B85-B89, B92, B94, B97, B99, K02, K04, K05, K61, N70-N74, P35, P37, P39, U00, Y95
		1B09	Fever of unknown origin	R50
	1C		**Perinatal conditions**	**P00-P03,P05,P07,P10-P15,P20-P22,P24-P29,P50,P52,P61,P77,P90-P91**
		1C01	Prematurity & low birthweight	P01, P05, P07, P22, P25-P28, P52, P61, P77
		1C02	Birth asphyxia & birth trauma	P00, P02-P03. P10-P15, P20-P21, P24, P29, P50, P90-P91
	1D		**Other conditions**	**D50-D53,E00-E02,E40-E46,E50-E56,E59-E61,E63-E64,Q00-Q07,Q10-18,Q20-Q28,Q30-Q45,Q50-56,Q60-Q87,Q89-Q93,Q95-Q99,X53-X54**
		1D01	Nutritional diseases	D50-D53, E00-E02, E40-E46, E50-E56, E59-E61, E63-E64, X53-X54
		1D02	Congenital anomalies	Q00-Q07, Q10-18, Q20-Q28, Q30-Q45, Q50-56, Q60-Q87, Q89-Q93, Q95-Q99
2			**NonCommunicable diseases**	
	2A		**Noncommunicable diseases**	**C00-C97,D00-D48,D55-D89,E03-E35,E65-E90,F00-F99,G10-G99,H00-H06,H11-H59,H61-H62,H69,H72-H95,I00-I28,I31,I34-I38,I42-I99,J30-J31,J33-J35,J37-J47,J60,J64,J66-J70,J80-J82,J84,J90-J99,K00-K01,K03,K06-K60,K62-K63,K70-K80,K82-K93,L05,L10-L99,M02-M54,M61-M85,M87-M99,N00-N08,N11-N29,N31-N33,N35-N40,N42-N48,N50-N51,N60,N62-N64,N75-N99,P04,P08,P51,P53-P60,P70-P72,P74-P76,P78,P80-P83,P92-P94,R00-R01,R03-R05,R06,R10-R23,R26,R27,R29-R49,R55-R56,R59,R63,R70-R74,R76-R77,R80-R82,R84-R87,R90-R91**
		2A01	Noncommunicable diseases	C00-C97, D00-D48, D55-D89, E03-E35, E65-E90, F00-F99, G10-G99, H00-H06, H11-H59, H61-H62, H69, H72-H95, I00-I28, I31, I34-I38, I42-I99, J30-J31, J33-J35, J37-J47, J60, J64, J66-J70, J80-J82, J84, J90-J99, K00-K01,K03, K06-K60, K62-K63, K70-K80, K82-K93, L05, L10-L99, M02-M54, M61-M85, M87-M99, N00-N08, N11-N29, N31-N33, N35-N40, N42-N48, N50-N51, N60, N62-N64, N75-N99, P04, P08, P51,P53-P60, P70-P72, P74-P76, P78, P80-P83, P92-P94, R00-R01, R03-R05, R06, R10-R23, R26, R27, R29-R49, R55-R56, R59, R63,R70-R74, R76-R77, R80-R82, R84-R87, R90-R91
3			**Injuries**	
	3A		**Injuries**	**V01-V99, W00-W99, X00-X52, X57-X99, Y00-Y91, Y97-Y98**
		3A01	Injuries ±	V01-V99, W00-W99, X00-X52, X57-X99, Y00-Y91, Y97-Y98
4			**Ill-defined conditions**	
	4A		**Ill defined or cause unknown**	**P96, R02, R07, R09, R25, R51-R54, R57-R58, R60-R62, R64, R68-R69, R78-R79, R83, R89, R92-R99**
		4A01	Ill defined or cause unknown	P96, R02, R07, R09, R25, R51-R54, R57-R58, R60-R62, R64, R68-R69, R78-R79, R83, R89, R92-R99

± External causes of injuries exclude S00-S99,T00-T99

MDS neonatal cause of death classification (ages 0-28 days)

NCodex4	NCodex2	NCodex	Disease	ICD 10 range
1			Communicable, perinatal and nutritional conditions	
	1A		Neonatal_Infections:	A20-A28, A32,A37-A44-A49,A68-A70,A74-A79,A81-A89,B95-B96,G00-G09,H10,H60,H65-H68,H70-H71,I30,I32-I33,I39-I41,J00-J22,J32,J36,J85-J86,K65,K67,K81,L00-L04,L08,M00-M01,M60,M86,N10,N30,N34,N41,N49,N61,P23,P35-P39,U04
		1A01	Neonatal Pneumonia	A37, H65-H68, H70, H71, J00-J22, J32, J36, J85, J86, P23, U04
		1A02	Sepsis	A20-A28, A32, A38, A40-A44, A46, A48, A49, A68-A70, A74, A75, A77-A79, B95, B96, H10, H60, I30, I32-I33, I39-I41, K65, K67, K81, L00-L04,L08, M00-M01, M60, M86, N10, N30, N34, N41, N49, N61, P35-P39
		1A03	Meningitis/encephalitis	A39, A81-A89, G00-G09
	1B		Other communicable diseases	A00-A09,A15-A19,A30-A31,A33-A36,A50-A67,A71,A80,A90-A99,B00-B09,B15-B27,B30,B33-B60,B64-B83,B85-B92,B94,B97,B99,J65,K04-K05,K61,N70-N74,R50,R75,U00,Y95
		1B01	Diarrhoeal diseases	A00-A09
		1B02	Tetanus	A33-A35
		1B03	Poliomyelitis	A80, B91
		1B04	Measles	B01, B05
		1B05	Malaria	B50-B54
		1B06	Other infectious and parasitic diseases	A15-A19, A30-A31, A36, A50-A67, A71, A90-A99, B00, B02-B04, B06-B09, B15-B27, B30, B33-B49, B55--B60, B64-B83,B85-B90, B92, B94, B97, B99, J65, K04, K05, K61, N70-N74, R75, U00, Y95
		1B07	Fever of unknown origin	R50
	1C		Perinatal conditions	P00-P03,P05,P07,P10-P15,P20-P22,P24-P29,P50,P52,P61,P77,P90-P91,R04
		1C01	Prematurity & low birthweight	P01, P05, P07, P22, P25-P28, P52, P61, P77, R04
		1C02	Birth asphyxia & birth trauma	P00, P02, P03. P10-P15, P20, P21, P24, P29, P50, P90, P91
	1D		Other perinatal conditions	D50-D53,E00-E02,E40-E46,E50-E56,E59-E61,E63-E64,G10-G99,Q00-Q99,X53-X54
		1D01	Congenital anomalies	G10-G99, Q00-Q99
		1D02	Nutritional diseases	D50-D53, E00-E02, E40-E46, E50-E56, E59-E61, E63, E64, X53-X54
2			NnCommunicable diseases	
	2A		Noncommunicable diseases	C00-C97,D00-D48,D55-D89,E03-E35,E65-E90,F00-F99,H00-H06,H11-H59,H61-H62,H69,H72-H95,I00-I28,I31,I34-I38,I42-I99,J30-J31,J33-J35,J37-J47,J60,J64,J66-J70,J80-J82,J84,J90-J99,K00-K03,K06-K60,K62-K63,K70-K80,K82-K93,L05,L10-L99,M02-M54,M61-M85,M87-M99,N00-N08,N11-N29,N31-N33,N35-N40,N42-N48,N50-N51,N60,N62-N64,N75-N99,P04,P08,P51,P53-P60,P70-P72,P74-P76,P78,P80-P83,P92-P94,R00-R01,R03,R05-R06,R10-R23,R26-R27,R29-R36,R39-R49,R55-R56,R59,R63,R70-R74,R76-R77,R80-R82,R84-R87,R90-R91
		2A01	Noncommunicable diseases	C00-C97, D00-D48, D55-D89, E03-E35, E65-E90, F00-F99, H00-H06, H11-H59, H61-H62, H69, H72-H95, I00-I28, I31, I34-I38, I42-I99, J30, J31, J33-J35, J37-J47, J60, J64, J66-J70, J80-J82, J84, J90-J99, K00-K03, K06-K60, K62-K63, K70-K80, K82-K93, L05, L10-L99, M02-M54, M61-M85, M87-M99, N00-N08, N11-N29, N31-N33, N35-N40, N42-N48, N50-N51, N60, N62-N64, N75-N99, P04, P08, P51, P53-P60, P70-P72, P74-P76, P78, P80-P83, P92-P94, R00, R01, R03, R05, R06, R10-R23,R26, R27, R29-R36, R39-R49, R55, R56, R59, R63, R70-R74, R76-R77, R80-R82, R84-R87, R90, R91
3			Injuries	
	3A		Injuries	S00-S99,T00-T98,V01-V99,W00-W99,X00-X52,X57-X99,Y00-Y91,Y97-Y98
		3A01	Injuries	S00-S99, T00-T98, V01-V99, W00-W99, X00-X52, X57-X99, Y00-Y91, Y97-Y98
4			Ill-defined conditions	
	4A		Ill defined or cause unknown	P96,R02,R07,R09,R25,R51-R54,R57-R58,R60-R62,R64,R68,R69,R78,R79,R83,R89,R92-R99
		4A01	Ill defined or cause unknown	P96, R02, R07, R09,R25, R51-R54, R57-R58, R60-R62, R64, R68, R69, R78, R79, R83, R89, R92-R99

GBD cause of death classification

	GBD2010_Name	GBD2010_ICD10 Code
A	Communicable, maternal, neonatal and nutritional disorders	A00,A01,A02-A04,A06-A09,A15-A19,A20-A28,A30,A31-A32,A33-A35,A36,A37,A38,A39,A42-A49(exceptA48.0,A48.3,A49.9),A50-A58,A59**,A60**,A63**,A65-A70(exceptA68),A71**,A74(exceptA74.0),A74.0**,A80,A81,A82,A83-A86,A87,A88-A89,A90-A91,A95,A99,B01-B02,B03,B04,B05,B06,B15,B16,B17.0,B17.1,B17.2,B20-B24,B25,B26,B27-B49(exceptB35-B36),B50-B54,B55,B56,B57,B58-64,B65,B66,B67,B69,B73,B74(exceptB74.3,B74.4,B74.8,B74.9),B76,B90,B92,B94.0**,B94.1,C46-C469,D50,D51-D53,D64.9,D84.9,E00-E02,E40-E46,E50,E51-E63.9,E64.0,64.1,E64.2-E64.3,G00.0,G00.1,G00.2-G03.9,G04,H65-H68,H70,J00-J06,J09-J11,J12(exceptJ12.1),J12.1,J13,J14,J15-J22,J85,N70-N73.9,O00-O99,P00,P01.0,P01.1,P01.2-P01.6,P01.7-P01.9,P02-P03,P04,P05,P07,P08.0-P08.2,P10-P15,P20-P21,P22,P23,P24,25-P28,P29,P35.0,P35.1,P35.2,P35.3,P35.3,P35.8,P35.8,P35.9,P36,P37.0,P37.2,P37.3,P37.4,P37.5-P37.9,P38-P39,P50-P74(exceptP61.2,P52,P52,P61.2,P70,P70),P76,P77,P78-P83,P90-P91,P92-P94,P96(exceptP96.9)
A.1	HIV/AIDS and tuberculosis	A15-A19, B90, P37.0, B20-B24, C46-C469, D84.9
A.1.1	Tuberculosis	A15-A19, B90, P37.0
A.1.2	HIV/AIDS	B20-B24, C46-C469, D84.9
A.1.2.1	HIV disease resulting in mycobacterial infection	B20.0
A.1.2.2	HIV disease resulting in other specified or unspecified diseases	B20-B24(except B20.0), C46-C46.9, D84.9
A.2	Diarrhea, lower respiratory infections, meningitis, and other common infectious diseases	A00, A02-A04, A06-A09, A01, J09-J11, J13, J14, J12.1, J12 (except J12.1), J15-J22, J85, P23, J00-J06, H65-H68, H70, G00.1, G00.0, A39, A87, G00.2-G03.9, A83-A86, B94.1, G04, A36, A37, A33-A35, B05, B01-B02, P35.8
A.2.1	Diarrheal diseases	A00, A02-A04, A06-A09
A.2.1.1	Cholera	A00
A.2.1.2	Other salmonella infections	A02
A.2.1.3	Shigellosis	A03
A.2.1.4	Enteropathogenic E coli infection	A04.0
A.2.1.5	Enterotoxigenic E coli infection	A04.1
A.2.1.6	Campylobacter enteritis	A04.5
A.2.1.7	Amoebiasis	A06
A.2.1.8	Cryptosporidiosis	A07.2
A.2.1.9	Rotaviral enteritis	A08.0
A.2.1.10.	Other diarrheal diseases	A04 (except A04.0, A04.1, A04.5), A07-A09 (except A07.2, A08.0), A05
A.2.2	Typhoid and paratyphoid fevers	A01
A.2.3	Lower respiratory infections	J09-J22, J85, P23
A.2.3.1	Influenza	J09-J11
A.2.3.2	Pneumococcal pneumonia	J13
A.2.3.3	H influenza type B pneumonia	J14
A.2.3.4	Respiratory syncytial virus pneumonia	J12.1
A.2.3.5	Other lower respiratory infections	J12 (except J12.1), J15-J22, J85, P23
A.2.4	Upper respiratory infections	J00-J06
A.2.5	Otitis media	H65-H68, H70
A.2.6	Meningitis	G00.1, G00.0, A39, A87, G00.2-G03.9
A.2.6.1	Pneumococcal meningitis	G00.1
A.2.6.2	H influenza type B meningitis	G00.0
A.2.6.3	Meningococcal infection	A39
A.2.6.4	Other meningitis	A87, G00.2-G03.9
A.2.7	Encephalitis	A83-A86, B94.1, G04
A.2.8	Diphtheria	A36
A.2.9	Whooping cough	A37
A.2.10	Tetanus	A33-A35
A.2.11	Measles	B05
A.2.12	Varicella	B01-B02, P35.8
A.3	Neglected tropical diseases and malaria	B50-B54, P37.3, P37.4, B57, B55, B56, B65, B69, B67, B74 (except B74.3, B74.4, B74.8, B74.9)B73, A71**, A74.0**, B94.0**A90-A91, A95, A82, B76, B66
A.3.1	Malaria	B50-B54, P37.3, P37.4
A.3.2	Chagas disease	B57
A.3.3	Leishmaniasis	B55
A.3.4	African trypanosomiasis	B56
A.3.5	Schistosomiasis	B65
A.3.6	Cysticercosis	B69
A.3.7	Echinococcosis	B67
A.3.8	Lymphatic filariasis	B74 (except B74.3, B74.4, B74.8, B74.9)
A.3.9	Onchocerciasis	B73
A.3.10	Trachoma	A71**, A74.0**, B94.0**
A.3.11	Dengue	A90-A91
A.3.12	Yellow fever	A95
A.3.13	Rabies	A82
A.3.14	Intestinal nematode infections	B77, B79, B76, B68, B70-B71, B78, B80-B81
A.3.15	Food-borne trematodiases	B66

A.3.16		Other neglected tropical diseases	A68, A69.2, A75-A79, A92-A94, A96, A98, B58- B64, B72, B74.3-B74.9, B83, P37.1, B70-B71, B78, B80-B81
**A.4**		**Maternal disorders**	**O00-O99**
A.4.1		Maternal hemorrhage	O20, O44-O46, O67, O72
A.4.2		Maternal sepsis	O85-O86
A.4.3		Hypertensive disorders of pregnancy	O10-O16
A.4.4		Obstructed labor	O64-O66
A.4.5		Abortion	O00-O08
A.4.6		Other maternal disorders	O21-O43, O47-O63, O68-O71, O73-O84, O87-O92, O94- O99
**A.5**		**Neonatal disorders**	**P01.0, P01.1, P07, P22, P25-P28, P61.2, P77, P52, P00, P01.7-P01.9 , P02-P03 , P08.0-P08.2, P10-P15, P20-P21, P24, P70, P90-P91, P36, P38-P39, P01.2-P01.6, P04, P05, P29, P50-P74(except P61.2, P52, P70), P76, P78-P83, P92- P94, P96(except P96.9)**
A.5.1		Preterm birth complications	P01.0, P01.1, P07, P22, P25-P28, P61.2, P77, P52
A.5.2		Neonatal encephalopathy (birth asphyxia and birth trauma)	P00, P01.7-P01.9 P02-P03 , P08.0-P08.2, P10-P15, P20-P21, P24, P70, P90-P91
A.5.3		Sepsis and other infectious disorders of the newborn baby	P36, P38-P39
A.5.4		Other neonatal disorders	P01.2-P01.6, P04, P05, P29, P50-P74(except P61.2, P52, P70), P76, P78-P83, P92-P94, P96(except P96.9)
**A.6**		**Nutritional deficiencies**	**D50-D53,,D64.9,E00-E02,E40-E46, E51-E63.9, E64.0, E64.1, E64.2-E64.3,E50**
A.6.1		Protein-energy malnutrition	E40-E46, E64.0
A.6.2		Iodine deficiency	E00-E02
A.6.3		Vitamin A deficiency	E50, E64.1
A.6.4		Iron-deficiency anemia	D50, D64.9
A.6.5		Other nutritional deficiencies	D51-D53, E51-E63.9, E64.2-E64.3
**A.7**		**Other communicable, maternal, neonatal and nutritional disorders**	**A20-A28,A30, A31- A32, A38, A42-A49 (except A48.0, A48.3, A49.9), A50-A58, A59**, A60**, A63**B15, A65- A70(except A68), A74 (except A74.0), A80, A81, A88-A89, A99, B03, B04, B06, B16, B17.0, B17.1, B17.2, B25, B26, B27-B49(except B35-B36), B58-B64, B91, B92, N70-N73.9, P35.0, P35.1, P35.2, P35.3, P35.3, P35.8, P35.9, P37.2, P37.5-P37.9**
A.7.1		Sexually transmitted diseases excluding HIV	N70-N73.9, A50-A58, A59**, A60**, A63**
	A.7.1.1	Syphilis	A50-A53
	A.7.1.2	Sexually transmitted chlamydial diseases	A55-A56
	A.7.1.3	Gonococcal infection	A54
	A.7.1.4	Trichomoniasis	A59**
	A.7.1.5	Other sexually transmitted diseases	A57-A58, A60**, A63**
A.7.2		Hepatitis	B15, B16, B17.0, P35.3, P35.3, B17.1, B17.2
	A.7.2.1	Acute hepatitis A	B15
	A.7.2.2	Acute hepatitis B	B16, B17.0, P35.3
	A.7.2.3	Acute hepatitis C	B17.1
	A.7.2.4	Acute hepatitis E	B17.2
A.7.3		Leprosy	A30, B92
A.7.4		Other infectious diseases	A20-A28, A31-A32, A38, A42-A49 (except A48.0, A48.3, A49.9),A65-A70(except A68), A74 (except A74.0), A81, A88-A89, A99, B04, B25, B27-B49(except B35-B36), B58-B64, P35.1, P35.2, P35.8, P35.9, P37.2, P37.5-P37.9, B03, B26, B06, P35.0, A80, B91
**B**		**Non-communicable diseases**	**B07**-B09**,L70**,L63.0**, B18, B35**, B36.0**, B36.1**, B36.2**, B36.3**, B36.8**, B36.9** B00, B66, B85**, B87**, B88**, C09-C13, C15-C15.9, C17, C22, C23, C24, C25, C30-C31, C33-C34, C37-C41, C43, C45, C47-C49, C51-C52, C57-C60(except C57.9), C63(except C63.9), C68- C69, C74-C75 (except C75.9), C77-C79, C82-C85, C88- C90, C91-C95, C96, C97, D0.5-D05.9*, D00.1*C16, D00.2*, D00-D24, D01.0-D01.3*, D01.4*, D01.5*, D02.0*, D02.1-D02.2*, D03*, D04*C56, D06*C54, D07.0, D07.1-D07.4*, D07.5*, D09.0*, D09.1-D09.3*, D25, D26- D44.2, D37.1*, D37.2*, D37.3- D37.5*C00-C08, D37.6*C32, D37.7*, D38.0*, D38.1*C50, D38.2- D38.5*, D39.0*C61, D39.1*C62, D39.2-D39.7*, D40.0*C18-C21, D40.1*C64- C66, D40.7*, D41.0-D41.2*C67, D41.4*C70-C72, D41.7*, D42- D43.9*, D44.0*C81, D44.1-D44.2*, D44.3-D44.5*C73, D44.6-D44.9*, D44.6-D48.9**I01, D48.0-D48.4*, D48.5*C44, D48.6*C53, D48.7*, D55-D59, D60-D64(except D64.9), D66-D89 (except D84.9, D86.0, D86.0, D86.2, D86.2, D86.9), D86.9, E03-E07, E10.2, E11.2, E11.2, E12.2, E12.2, E13.2), E13.2, E15-E34, E28.2, E65-E67, E70-E85 (except E85.3, E85.4, E85.8, E85.9), E88 (except E88.9), E89-E96, F00-F03, F04-F09, F10, F11, F12, F13, F14, F15, F16, F17, F18-F19, F20-F29, F32**-F33**, F34.1**F30**-F31**F40**- F42**, F34-F39 (except F34.1), F43**-F44**F50, F45-F48**, F51-F53**, F54-F59, F60-F69**, F70**- F79**, F70**-F79**, F80-F84** (except F84.0, F84.5, F84.9), F88-F89**E10- E13 (except E10.2, F90**, F91**-F92**, F93-F98**, G06-G08, G10-G12, G20-G21, G23-G25, G30-G31, G35, G36-G37, G40-G41, G43**, G44**, G45, G47-G58**, G60-G72, G90-G91 (except G91.1, G91.3, G91.8), G93.2, G93.3, G93.4, G93.5, G93.6), G93-G98 (except G93.1, H00-H21**, H25- H26**, H27*, H30-H35** (except H35.3), H35.3**, H40**, H43-H47**, H49-H52**, H53-H54**, H55- H61**, H68-H69**, H71-H83**, H90-H91**, H92- H93**, I00, I02.0, I02.9, I05-I09, I11, I12.0, I12.9, I13.1, I13.2, I13.9, I20-I25, I27-I28 (except I27.1), I30-I32(except I31.2, I31.3), I33, I34-I39, I40, I42, I47, I48, I60-I63 I65-I67, I69.0, I69.1, I69.2, I69.3, I70.2, I70.8, I71, I72, I73, I77-I80, I82-I84, I85, I85, I86- I98, J30-J39, J40-J44, J45-J46, J47, J60-J65, J66-J70(except J69), J82, J84, J92, J93.0, J93.1, J95, J98 (except J98.1, J98.2, J98.3, J98.9)B18, K00-K01**, K02**, K03-K04**, K05**, K06-K11**, K12-K13, K14**, K20-K22, K25-K27, K28, K29, K30-K31, K35-K37, K38, K40-K41, K50-K52, K55, K56, K57-K63, K66.8, K70, K70, K71.7, K71.7, K72.1-K72.9, K72.1-K72.9, K73- K74, K73-K74, K75.2-K75.9, K75.2-K75.9, K76.6- K76.7, K76.6-K76.7, K76.9, K76.9, K760-K765, K768, K77, K80-K83, K85- K86.9, K90-K92 (except K92.0, K92.1, K92.2, K92.9), L00, L01, L02, L03.0, L03.1**, L03.2- L03.9, L04**, L05.0, L05.9, L08, L10-L13, L28**, L29**, L30**, L40**-L41**, L42**-L44**, L50**L89, L51, L52**-L53**, L55**-L60**, L63.1**, L63.8**, L63.9**, L64**-L68**, L71**-L75**, L80**-L85**, L87, L88, L90**-L92**, L93, L94-L95, L97, L98.0-L98.4**, M00-M02, M05-M06, M08, M10**, M11**, M12-M13, M15-M19**, M20-M25**, M30-M35, M40**, M41-M46 (except M46.9), M46.9, M47**, M48** (except M48.0, M48.0-M48.2**, M48.1, M48.2, M48.8**- M48.9**, M48.8, M48.9), M50-M54**, M60**, M61-M62, M65-M71**, M72, M75- M79**, M80-M85, M87-M94, M95-M99**, N00-N01, N02- N07, N10-N12, N13, N15.0, N15.1-N15.9, N20-N28, N30-N32, N34, N35-N36, N39.0, N39.1-N39.9, N40**, N41-N45, N46**, N47-N50, N80, N81, N94.3, N97**, Q00, Q01-Q02, Q03, Q04, Q05, Q06-Q18, Q20-Q28, Q30-Q34, Q35-Q37, Q38-Q45, Q50-Q89 (except Q89.9)L20**-L28**, Q90, Q91-Q99 (except Q99.9), R95, X45**
**B.1**		**Neoplasms**	**C15-C159, D00.1*C16, D00,2*, D37.1*, C22, D01.5*, D37.6*C32, D02.0*, D38.0*, C33-C34 , D02.1-D02.2*, D38.1*C50, D0.5-D05.9*, D48.6*C53, D06*C54, D07.0, D39.0*C61, D07.5*, D40.0*C18-C21, D01.0-D01.3*, D37.3- D37.5*C00-C08, C11, C09-C10, C12-C13, C23, C24, C25, C43, D03*, D48.5*C44, D04*C56, D39.1*C62, D40.1*C64- C66, D41.0-D41.2*C67, D09.0*, D41.4*C70-C72, D42- D43.9*, D44.3-D44.5*C73, D44.0*C81, C82-C85, C96, C88- C90, C91-C95, C17, C30-C31, C37-C41, C45, C47-C49, C51-C52, C57-C60(except C57.9), C63(except C63.9), C68- C69, C74-C75 (except C75.9), C77-C79, C97, D01.4*, D07.1-D07.4*, D09.1-D09.3*, D37.2*, D37.7*, D38.2- D38.5*, D39.2-D39.7*, D40.7*, D41.7*, D44.1-D44.2*, D44.6-D44.9*, D48.0-D48.4*, D48.7*, D00-D24, D26- D44.2, D44.6-D48.9***
B.1.1		Esophageal cancer	C15-C159, D00.1*
B.1.2		Stomach cancer	C16, D00,2*, D37.1*
B.1.3		Liver cancer	C22, D01.5*, D37.6*

B.1.3.1	Liver cancer secondary to hepatitis B	
B.1.3.2	Liver cancer secondary to hepatitis C	
B.1.3.3	Liver cancer secondary to alcohol use	
B.1.3.4	Other liver cancer	
B.1.4	Larynx cancer	C32, D02.0*, D38.0*
B.1.5	Trachea, bronchus and lung cancers	C33-C34 , D02.1-D02.2*, D38.1*
B.1.6	Breast cancer	C50, D0.5-D05.9*, D48.6*
B.1.7	Cervical cancer	C53, D06*
B.1.8	Uterine cancer	C54, D07.0, D39.0*
B.1.9	Prostate cancer	C61, D07.5*, D40.0*
B.1.10	Colon and rectum cancers	C18-C21, D01.0-D01.3*, D37.3-D37.5*
B.1.11	Mouth cancer	C00-C08
B.1.12	Nasopharynx cancer	C11
B.1.13	Cancer of other part of pharynx and oropharynx	C09-C10, C12-C13
B.1.14	Gallbladder and biliary tract cancer	C23, C24
B.1.15	Pancreatic cancer	C25
B.1.16	Malignant melanoma of skin	C43, D03*, D48.5*
B.1.17	Non-melanoma skin cancer	C44, D04*
B.1.18	Ovarian cancer	C56, D39.1*
B.1.19	Testicular cancer	C62, D40.1*
B.1.20	Kidney and other urinary organ cancers	C64-C66, D41.0-D41.2*
B.1.21	Bladder cancer	C67, D09.0*, D41.4*
B.1.22	Brain and nervous system cancers	C70-C72, D42-D43.9*, D44.3-D44.5*
B.1.23	Thyroid cancer	C73, D44.0*
B.1.24	Hodgkin's disease	C81
B.1.25	Non-Hodgkin's lymphoma	C82-C85, C96
B.1.26	Multiple myeloma	C88-C90
B.1.27	Leukemia	C91-C95
B.1.28	Other neoplasms	C17, C30-C31, C37-C41, C45, C47-C49, C51-C52, C57- C60(except C57.9), C63(except C63.9), C68-C69, C74-C75 (except C75.9), C77-C79, C97, D00-D24, D01.4*, D07.1-D07.4*, D09.1-D09.3*, D26-D44.2, D37.2*, D37.7*, D38.2-D38.5*, D39.2- D39.7*, D40.7*, D41.7*, D44.1-D44.2*, D44.6- D48.9**, D44.6-D44.9*, D48.0-D48.4*, D48.7*
**B.2**	**Cardiovascular and circulatory diseases**	**G45, I00, I01, I02.0, I02.9, I05-I09, I11, I20-I25, I27-I28 (except I27.1), I30-I32(except I31.2, I31.3), I33, I34-I39, I40, I42, I47, I48, I60-I63 I65-I67, I69.0, I69.1, I69.2, I69.3, I70.2, I70.8, I71, I72, I73, I77-I80, I82-I84, I86-I98**
B.2.1	Rheumatic heart disease	I01, I02.0, I05-I09
B.2.2	Ischemic heart disease	I20-I25
B.2.3	Cerebrovascular disease	I60-I63 I65-I67, I69.0, I69.1, I69.2, I69.3
B.2.3.1	Ischemic stroke	I63, I65-I67(except I67.4), I69.3
B.2.3.2	Hemorrhagic and other non- ischemic stroke	I60-I62, I69.0-I69.2, I67.4
B.2.4	Hypertensive heart disease	I11
B.2.5	Cardiomyopathy and myocarditis	I42, I40
B.2.6	Atrial fibrillation and flutter	I48
B.2.7	Aortic aneurysm	I71
B.2.8	Peripheral vascular disease	I73, I70.2
B.2.9	Endocarditis	I33
B.2.10	Other cardiovascular and circulatory diseases	I00,I02.9,I27-I28 (except I27.1),I30-I32(except I31.2, I31.3), I34-I39, I47, I70.8, I72, I77-I80, I82-I84, I86-I98, G45
**B.3**	**Chronic respiratory diseases**	**B18, D86.0, D86.2, D86.9, I85, J40-J44, J45-J46, J47J, J60-J65, J66-J70(except J69), J82, J84J30- J39, J92, J93.0, J93.1, J95, J98 (except J98.1, J98.2, J98.3, J98.9), K70, K71.7, K72.1-K72.9, K73-K74, K75.2-K75.9, K76.6-K76.7, K76.9**
B.3.1	Chronic obstructive pulmonary disease	J40-J44, J47
B.3.2	Pneumoconiosis	J60-J65
B.3.3	Asthma	J45-J46
B.3.4	Interstitial lung disease and pulmonary sarcoidosis	D86.0, D86.2, D86.9, J84
B.3.5	Other chronic respiratory diseases	J30-J39, J66-J70(except J69), J82, J92, J93.0, J93.1, J95, J98 (except J98.1, J98.2, J98.3, J98.9)
**B.4**	**Cirrhosis of the liver**	**B18, I85, K70, K71.7, K72.1-K72.9, K73-K74, K75.2-K75.9, K76.6-K76.7, K76.9**
B.4.1	Cirrhosis of the liver secondary to hepatitis B	
B.4.2	Cirrhosis of the liver secondary to hepatitis C	
B.4.3	Cirrhosis of the liver secondary to alcohol use	
B.4.4	Other cirrhosis of the liver	
**B.5**	**Digestive diseases (except cirrhosis)**	**K25-K27, K29, K35-K37, K56, K40-K41, K50-K52, K55, K80-K83, K85-K86.9, K00-K01**, K03-K04**, K06-K11**, K12-K13, K14**, K20-K22, K28, K30-K31, K38, K57-K63, K66.8, K760-K765, K768, K77, K90-K92 (except K92.0, K92.1, K92.2, K92.9)**
B.5.1	Peptic ulcer disease	K25-K27
B.5.2	Gastritis and duodenitis	K29
B.5.3	Appendicitis	K35-K37
B.5.4	Paralytic ileus and intestinal obstruction without hernia	K56
B.5.5	Inguinal or femoral hernia	K40-K41
B.5.6	Noninfective inflammatory bowel disease	K50-K52
B.5.7	Vascular disorders of intestine	K55

B.5.8		Gall bladder and bile duct disease	K80-K83
B.5.9		Pancreatitis	K85-K86.9
B.5.10		Other digestive diseases	K00-K01**, K03-K04**, K06-K11**, K12-K13, K14**, K20-K22, K28, K30-K31, K38, K57-K63, K66.8, K760- K765, K768, K77, K90-K92 (except K92.0, K92.1, K92.2, K92.9)
**B.6**		**Neurological disorders**	**F00-F03, G30-G31, G20-G21, G40-G41, G35, G43**, G44**, G06-G08, G10-G12, G23-G25, G36-G37, G47- G58**, G60-G72, G90-G91 (except G91.1, G91.3, G91.8), G93-G98 (except G93.1, G93.2, G93.3, G93.4, G93.5, G93.6)**
B.6.1		Alzheimer's disease and other dementias	F00-F03, G30-G31
B.6.2		Parkinson's disease	G20-G21
B.6.3		Epilepsy	G40-G41
B.6.4		Multiple sclerosis	G35
B.6.5		Migraine	G43**
B.6.6		Tension-type headache	G44**
B.6.7		Other neurological disorders	G06-G08, G10-G12, G23-G25, G36-G37, G47-G58**, G60- G72, G90-G91 (except G91.1, G91.3, G91.8), G93-G98 (except G93.1, G93.2, G93.3, G93.4, G93.5, G93.6)
**B.7**		**Mental and behavioral disorders**	**F20-F29, F10, X45, Q86.0, F11, F14, F15, F12, F13, F16, F18-F19, X41, X42,X49, F32**-F33**, F34.1**F30**- F31**, F40**-F42**, F43**-F44**F50, F90**, F91**-F92**, F70**-F79**, F70**-F79**, F04-F09, F17, F34-F39 (except F34.1), F45-F48**, F51-F53**, F54-F59, F60-F69**, F80- F84** (except F84.0, F84.5, F84.9), F93-F98**, F88-F89****
B.7.1		Schizophrenia	F20-F29
B.7.2		Alcohol use disorders	F10, X45, Q86.0
B.7.3		Drug use disorders	F11, F14, F15, F12, F13, F16, F18-F19, X41, X42, X49
	B.7.3.1	Opioid use disorders	F11
	B.7.3.2	Cocaine use disorders	F14
	B.7.3.3	Amphetamine use disorders	F15
	B.7.3.4	Cannabis use disorders	F12
	B.7.3.5	Other drug use disorders	F13, F16, F18-F19
B.7.4		Unipolar depressive disorders	F32**-F33**, F34.1**
	B.7.4.1	Major depressive disorder	F32**-F33**
	B.7.4.2	Dysthymia	F34.1**
B.7.5		Bipolar affective disorder	F30**-F31**
B.7.6		Anxiety disorders	F40**-F42**, F43**-F44**
B.7.7		Eating disorders	F50
B.7.8		Pervasive development disorders	F84.0**, F84.5**
	B.7.8.1	Autism	F84.0**
	B.7.8.2	Asperger's syndrome	F84.5**
B.7.9		Childhood behavioral disorders	F90**, F91**-F92**, F70**-F79**
	B.7.9.1	Attention-deficit hyperactivity disorder	F90**
	B.7.9.2	Conduct disorder	F91**-F92**
B.7.10		Idiopathic intellectual disability	F70**-F79**
B.7.11		Other mental and behavioral disorders	F04-F09, F17, F34-F39 (except F34.1), F45-F48**, F51- F53**, F54-F59, F60-F69**, F80-F84** (except F84.0, F84.5, F84.9), F93-F98**, F88-F89**
**B.8**		**Diabetes, urogenital, blood, and endocrine diseases**	**D55-D59, D60-D64(except D64.9), D66-D89 (except D84.9, D86.0, D86.2, D86.9), E03-E07, E10.2, E10-E13 (except E10.2, E11.2, E11.2, E12.2, E12.2, E13.2), E13.2, E15-E34, E28.2, E65-E67, E70-E85 (except E85.3, E85.4, E85.8, E85.9), E88 (except E88.9), E89-E96, I12.0, I12.9, I13.1, I13.2, I13.9, N00-N01, N02-N07, N10-N12, N13, N15.0, N15.1-N15.9, N20-N23, N24-N28, N30, N31-N32, N34, N35-N36, N39.0, N39.1-N39.9, N40**, N41-N45, N46**, N47-N50, N80, N81, N94.3, N97****
B.8.1		Diabetes mellitus	E10-E13 (except E10.2, E11.2, E12.2, E13.2)
B.8.2		Acute glomerulonephritis	N00-N01
B.8.3		Chronic kidney diseases	E10.2, E11.2, E12.2, E13.2, I12.0, I12.9, I13.1, I13.2, I13.9, N02-N07, N15.0
	B.8.3.1	Chronic kidney disease due to diabetes mellitus	E10.2, E11.2, E12.2, E13.2, E14.2
	B.8.3.2	Chronic kidney disease due to hypertension	I12.0, I12.9, I13.0, I13.1, I13.2, I13.9
	B.8.3.3	Chronic kidney disease unspecified	N02-N07, N15.0
B.8.4		Urinary diseases and male infertility	N10-N12, N15.1-N15.9, N30, N34, N39.0,N20-N23, N40**, N46**, N13, N24-N28, N31-N32, N35-N36, N39.1-N39.9, N41-N45, N47-N50
	B.8.4.1	Tubulointerstitial nephritis, pyelonephritis, and urinary tract infect	N10-N12, N15.1-N15.9, N30, N34, N39.0
	B.8.4.2	Urolithiasis	N20-N23
	B.8.4.3	Benign prostatic hyperplasia	N40**
	B.8.4.4	Male infertility	N46**
	B.8.4.5	Other urinary diseases	N13, N24-N28, N31-N32, N35-N36, N39.1-N39.9, N41- N45, N47-N50
B.8.5		Gynecological diseases	D25, E28.2, N97**, N80, N81, N94.3
	B.8.5.1	Uterine fibroids	D25
	B.8.5.2	Polycystic ovarian syndrome	E28.2
	B.8.5.3	Female infertility	N97**
	B.8.5.4	Endometriosis	N80
	B.8.5.5	Genital prolapse	N81
	B.8.5.6	Premenstrual syndrome	N94.3
	B.8.5.7	Other gynecological diseases	N60**, N61-N64, N75-N76, N82-N83, N84**-N92 , N93- N94(Except N94.3), N98, N46**
B.8.6		Hemoglobinopathies and hemolytic anemias	D55-D59
	B.8.6.1	Thalassemias	D56

B.8.6.2	Sickle cell disorders	D57
B.8.6.3	G6PD deficiency	D55
B.8.6.4	Other hemoglobinopathies and hemolytic anemias	D58-D64.8
B.8.7	Other endocrine, nutritional, blood, and immune disorders	D66-D89 (except D84.9, D86.0, D86.2, D86.9), E03-E07, E15-E34, E65-E67, E70-E85 (except E85.3, E85.4, E85.8, E85.9), E88 (except E88.9), E89-E96, D60-D64(except D64.9)
**B.9**	**Musculoskeletal disorders**	**M05-M06, M15-M19**, M46.9, M47**, M48.0-M48.2**, M48.8**-M48.9**, M50-M54**, M10**, M00-M02, M08, M11**, M12-M13, M20-M25**, M30-M35,M40**, M41- M46 (except M46.9), M48** (except M48.0, M48.1, M48.2, M48.8, M48.9), M60**,M61-M62, M65-M71**, M72, M75- M79**, M80-M85, M87-M94 , M95-M99****
B.9.1	Rheumatoid arthritis	M05-M06
B.9.2	Osteoarthritis	M15-M19**
B.9.3	Low back and neck pain	M46.9, M47** , M48.0-M48.2**, M48.8**-M48.9**, M50- M54**
B.9.3.1	Low back pain	M46.9, M47**, M48.0-M48.2**,M48.8**-M48.9**, M51- M54**(except M53.1** ,M53.2**)
B.9.3.2	Neck pain	M50**, M53.1** ,M53.2**
B.9.4	Gout	M10**
B.9.5	Other musculoskeletal disorders	M00-M02, M08, M11** ,M12-M13, M20-M25**,M30- M35,M40**, M41-M46 (except M46.9), M48** (except M48.0, M48.1, M48.2, M48.8, M48.9), M60**, M61-M62, M65-M71**, M72, M75-M79**, M80-M85, M87-M94, M95- M99**
**B.10.**	**Other non-communicable diseases**	**B00,B07**- B09**, B35**, B36.0**, B36.1**, B36.2**, B36.3**, B36.8**, B36.9**, B66, B85**, B87**, B88**, H00-H21**, H25- H26**, H27*, H30-H35** (except H35.3), H35.3**, H40**, H43-H47**, H49-H52**, H53-H54**, H55- H61**, H68-H69**, H71-H83**, H90-H91**, H92-H93**, K02**, K05**, L00, L01, L02, L03.0, L03.1**, L03.2-L03.9, L04**, L05.0, L05.9, L08, L10-L13, L20**-L28**,L28**, L29**, L30**, L40**-L41**, L42**-L44**, L50**, L51, L52**-L53**, L55**- L60**, L63.0**, L63.1**, L63.8**, L63.9**, L64**-L68**, L70**, L71**-L75**, L80**-L85**, L87, L88, L89, L90**-L92**, L93, L94-L95, L97, L98.0-L98.4**, Q00, Q01-Q02, Q03, Q04, Q05, Q06-Q18, Q20-Q28, Q30-Q34, Q35-Q37, Q38-Q45, Q50- Q89 (except Q89.9), Q90, Q91-Q99 (except Q99.9), R95**
B.10.1	Congenital anomalies	Q00-Q99 (except Q89.9, Q99.9)
B.10.1.1.	Neural tube defects	Q00, Q01, Q05
B.10.1.2.	Congenital heart anomalies	Q20-Q28
B.10.1.3.	Cleft lip and cleft palate	Q35-Q37
B.10.1.5.	Down's syndrome	Q90
B.10.1.6.	Other chromosomal abnormalities	Q91-Q99 (except Q99.9)
B.10.1.7.	Other congenital anomalies	Q01-Q03, Q04, Q06-Q18, Q30-Q34, Q38-Q45, Q50-Q89 (except Q89.9)
B.10.2	Skin and subcutaneous diseases	B00,B07**-B09**,L70**, L63.0**, B35**, B36.0**, B36.1**, B36.2**, B36.3**, B36.8**, B36.9** , B66, B85**, B87**, B88**, L00, L01, L02, L03.0, L03.1**, L03.2-L03.9, L04**, L05.0, L05.9, L08, L10-L13, L20**-L28**, L28**, L29**, L30**, L40**-L41**, L42**-L44**, L50**L89, L51, L52**-L53**, L55**-L60**, L63.1**, L63.8**, L63.9**, L64**-L68**, L71**-L75**, L80**-L85**, L87, L88, L90**-L92**, L93, L94-L95, L97, L98.0-L98.4**
B.10.2.1.	Eczema	L20**-L28**
B.10.2.2.	Psoriasis	L40**-L41**
B.10.2.3.	Cellulitis	L03.0, L03.1**, L03.2-L03.9
B.10.2.4.	Abscess, impetigo, and other bacterial skin diseases	L00, L01, L02, L04**, L08, L88, L97, L98.0-L98.4**
B.10.2.5.	Scabies	B66**
B.10.2.6.	Fungal skin diseases	B35**, B36.0**, B36.1**, B36.2**, B36.3**, B36.8**, B36.9**
B.10.2.7.	Viral skin diseases	B00, B07**-B09**
B.10.2.8.	Acne vulgaris	L70**
B.10.2.9.	Alopecia areata	L63.0**, L63.1**, L63.8**, L63.9**,
B.10.2.10	Pruritus	L29**
B.10.2.11	Urticaria	L50**
B.10.2.12	Decubitus ulcer	L89
B.10.2.13	Other skin and subcutaneous diseases	B85**, B87**,B88**, L05.0, L05.9, L10-L13, L28**, L30**, L42**-L44**, L51, L52**-L53**, L55**-L60**, L64**-L68**, L71**-L75**, L80**-L85**, L87, L90**- L92**, L93, L94-L95
B.10.3	Sense organ diseases	H40**, H25-H26**, H35.3**,H49-H52**, H90-H91**, H30- H35** (except H35.3), H53-H54**, H00-H21**, H27*, H43- H47**, H55-H61**, H68-H69**, H71-H83**, H92-H93**
B.10.3.1.	Glaucoma	H40**
B.10.3.2.	Cataracts	H25-H26**
B.10.3.3.	Macular degeneration	H35.3**
B.10.3.4.	Refraction and accommodation disorders	H49-H52**
B.10.3.5.	Other hearing loss	H90-H91**
B.10.3.6.	Other vision loss	H30-H35** (except H35.3), H53-H54**
B.10.3.7.	Other sense organ diseases	H00-H21**, H27*, H43-H47**, H55-H61**, H68-H69** , H71-H83**, H92-H93**
B.10.4	Oral disorders	K02**, K05**
B.10.4.1.	Dental caries	K02**
B.10.4.2.	Periodontal disease	K05**
B.10.4.3.	Edentulism	No ICD code**
B.10.5	Sudden infant death syndrome	R95
**C**	**Injuries**	**V01-V04, V06, V09, V10-V19, V20-V29, V30-V79, V80, V81, V82 V05, V83-V86, V87.2-V87.3, V88.2, V88.3, V90, V91, V92, V93-V98W00-W19, W21, W24-W31, W39, W44, W45- W46, W49-W52, W53-W64, W65-W74, W75-W99, X00-X19, X20-X29, X30-X39, X40, X43-X44 W32-W34, X46-X47, X48, X50-X58, X60-X67, X68, X69, X70, X71, X72- X74, X75, X76-X77, X78-X83, X85-X92, X93-X95, X96-X98, X99, Y00-Y08, Y35, Y36, Y40-Y84, Y85.0, Y88, Y89.0, Y89.1**
**C.1**	**Transport injuries**	**V01-V04, V06,V09, V10-V19, V20-V29, Y85.0, V30-V79, V87.2-V87.3, V80, V82, V05,V81, V83-V86, V88.2, V88.3 , V91, V93-V98**
C.1.1	Road injury	V01-V04, V06, V09, V10-V19, V20-V29, Y85.0, V30-V79, V87.2-V87.3, V80, V82
C.1.1.1	Pedestrian injury by road vehicle	V01-V04, V06,V09
C.1.1.2	Pedal cycle vehicle	V10-V19

	C.1.1.3	Motorized vehicle with two wheels	V20-V29
	C.1.1.4	Motorized vehicle with three or more wheels	V30-V79, V87.2-V87.3
	C.1.1.5	Road injury other	V80, V82
C.1.2		Other transport injury	V05,V81, V83-V86, V88.2, V88.3 , V91, V93-V98
**C.2**		**Unintentional injuries other than transport injuries**	**V90, V92, W00-W19, W21,W24-W31,W32-W34, W39, W44, W45-W46,W53-W64, W49-W52,W65-W74,W75-W99, X00-X19, X20-X29,X40, X43-X44,X46-X47, X48, X50-X58,Y40-Y84,Y88**
C.2.1		Falls	W00-W19
C.2.2		Drowning	V90, V92, W65-W74
C.2.3		Fire, heat and hot substances	X00-X19
C.2.4		Poisonings	X46-X47, X48, X40, X43-X44
C.2.5		Exposure to mechanical forces	W32-W34, W24-W31, W45-W46
	C.2.5.1	Mechanical forces (firearm)	W32-W34
	C.2.5.2	Mechanical forces (other)	W24-W31, W45-W46
C.2.6		Adverse effects of medical treatment	Y40-Y84, Y88
C.2.7		Animal contact	X20-X29, W53-W64
	C.2.7.1	Animal contact (venomous)	X20-X29
	C.2.7.2	Animal contact (non-venomous)	W53-W64
C.2.8		Unintentional injuries not classified elsewhere	W21, W39, W44, W49-W52, W75-W99, X50-X58
**C.3**		**Self-harm and interpersonal violence**	**X60-X83, X85-X99, Y00-Y08**
C.3.1		Self-harm	X60-X83
C.3.2		Interpersonal violence	X93-X95, X99, X85-X92, X96-X98, Y00-Y08
	C.3.2.1	Assault by firearm	X93-X95
	C.3.2.2	Assault by sharp object	X99
	C.3.2.3	Assault by other means	X85-X92, X96-X98, Y00-Y08
**C.4**		**Forces of nature, war, and legal intervention**	**X30-X39, Y36, Y89.1, Y35, Y89.0**
C.4.1		Exposure to forces of nature	X30-X39
C.4.2		Collective violence and legal intervention	Y36, Y89.1,Y35, Y89.0

Notes:

The format of this list is slightly different from the list originally published. We reorganized the order and introduced a new hierarchical code label to make easily use for comparison with other classification systems.

* These codes have been used just for calculation of mortality (YLL)

** These codes have been used just for calculation of non-fatal outcomes (YLD)

*** These codes are just usable for hospital data from the US

Reference: Christopher JL Murray et.al. GBD 2010: design, definitions, and metrics The Lancet - 15 December 2012 (Vol. 380, Issue 9859, Pages 2063-2066) DOI: 10.1016/S0140-6736(12)61899-6

WHO cause of death classification

Code	Verbal autopsy title	ICD-10 code (to ICD)	ICD-10 range
**Communicable diseases**			
	**VAs-01 Infectious and parasitic diseases**		
1.01	Sepsis	A41	A40-A41
1.02	Acute respiratory infection, including pneumonia	J22/J18	J00-J22
1.03	HIV/AIDS related death	B24	B20-B24
1.04	Diarrheal diseases	A09	A00-A09
1.05	Malaria	B54	B50-B54
1.06	Measles	B05	B05
1.07	Meningitis and encephalitis	G03;G04	A39; G00-G05
1.08	Tetanus Excludes: Neonatal tetanus VAs-10.05	A35 (obstetrical A34)	A33-A35
1.09	Pulmonary tuberculosis	A16	A15-A16
1.1	Pertussis	A37	A37
1.11	Haemorrhagic fever	A99	A90-A99
1.99	Other and unspecified infectious disease	B99	A17-A19;A20-A38;A42-A89; B00-B19;B25-B49;B55-B99
	**VAs-03 Nutritional and endocrine disorders**		
3.01	Severe anaemia	D64	D50-D64
3.02	Severe malnutrition	E46	E40-E46
3.03	Diabetes mellitus	E14	E10-E14
	**VAs-09 Pregnancy, childbirth and puerperium-related disorders**		
9.01	Ectopic pregnancy	O00	O00
9.02	Abortion-related death	O06	O03-O08
9.03	Pregnancy-induced hypertension	O13 (or O15 for eclampsia)	O10-O16
9.04	Obstetric haemorrhage	O46 (ante partum)<br>O72 (post partum)	O46; O67; O72
9.05	Obstructed labour	O66	O63-O66
9.06	Pregnancy-related sepsis	O75.3 (ante partum)<br>O85 (post partum)	O85; O75.3
9.07	Anaemia of pregnancy	O99	O99.0
9.08	Ruptured uterus	O71	O71
9.99	Other and unspecified maternal cause	O05	O01-O02;O20-O45;O47-O62;O68-O70;O73-O84:O86-O99
	**VAs-10 Neonatal causes of death**		
10.01	Prematurity	P07	P05-P07
10.02	Birth asphyxia	P21	P20-P22
10.03	Neonatal pneumonia	P23	P23-P25
10.04	Neonatal sepsis	P63	P36
10.05	Neonatal tetanus	A33	A33
10.06	Congenital malformation	Q89	Q00-Q99
10.99	Other and unspecified perinatal cause of death	P96	P00-P04; P08-P15; P26-P35; P37-P94; P96
	**VAs-11 Stillbirths**		
11.01	Fresh stillbirth	P95	P95
11.02	Macerated stillbirth	P95	P95
**Non-communicable diseases** (see Note 1)			
	**VAs-02 Neoplasms**		
2.01	Oral neoplasms	C06	C00-C06
2.02	Digestive neoplasms	C26	C15-C26
2.03	Respiratory neoplasms	C39	C30-C39
2.04	Breast neoplasms	C50	C50
2.05	Female reproductive neoplasms	C57	C51-C58
2.06	Male reproductive neoplasms	C63	C60-C63
2.99	Other and unspecified neoplasms	C80	C07-C14, C40-C49, C60-D48
	**VAs-04 Diseases of the circulatory system**		
4.01	Acute cardiac disease	I24 (acute ischemic)	I20-I25
4.02	Stroke	I64	I60-I69
4.03	Sickle cell with crisis	D57	D57
4.99	Other and unspecified cardiac disease	I99	I00-I09, I10-I15, I26-I52, I70-I99
	**VAs-05 Respiratory disorders**		
5.01	Chronic obstructive pulmonary disease (COPD)	J44	J40-J44
5.02	Asthma	J45 (J46)	J45-J46

	**VAs-06 Gastrointestinal disorders**		
6.01	Acute abdomen	R10	R10
6.02	Liver cirrhosis	K74	K70-K76
	**VAs-07 Renal disorders**		
7.01	Renal failure	N19	N17-N19
	**VAs-08 Mental and nervous system disorders**		
8.01	Epilepsy	G40	G40-G41
98	**Other and unspecified non-communicable disease**.(See Note 2)	R99	D55-D89; E00-E07; E15-E35;E50-E90;F00-F99; G06-G09;G10-G37; G50-G99; H00-H95; J30-J39;J47-J99; K00-K31;K35-K38;K40-K93; L00-L99; M00-M99; N00-N16;N20-N99; R00-R09;R11-R94
	**VAs-12 External causes of death** (See Note 3)		
12.01	Road traffic accident	V89	V01-V89
12.02	Other transport accident	V99	V90-V99
12.03	Accidental fall	W19	W00-W19
12.04	Accidental drowning and submersion	W74	W65-W74
12.05	Accidental exposure to smoke, fire and flames	X09	X00-X19
12.06	Contact with venomous animals and plants	X29	X20-X29
12.07	Accidental poisoning and exposure to noxious substance	X49	X40-X49
12.08	Intentional self-harm	X84	X60-X84
12.09	Assault	Y09	X85-Y09
12.1	Exposure to force of nature	X39	X30-X39
12.99	Other and unspecified external cause of death	X59	S00-T99; W20-W64;W75-W99; X50-X59; Y10-Y98
99	**Cause of death unknown**	R99	R95-R99

Notes

(1) This group covers all non-communicable conditions. Any infection of the systems that are listed in this section should be assigned to the suitable infectious disease category. Any maternal and perinatal condition should be assigned to the maternal and perinatal causes below.

(2) This group covers all non-communicable conditions that could not be assigned to another category in this section. There is a separate category for cases where the cause of death is unknown.

(4) The list of questions contains sub questions that allow for more specificity for accidents.

(3) The format of this list is slightly different from the list originally published. We reorganized the order and introduced a new hierarchical code label to make easily use for comparison with other classification systems.

Source: WHO verbal autopsy instrument (2012) Appendix 1: 2012 cause of death list for verbal autopsy with corresponding ICD-10 codes pp A1-1 to A1-6
