# Supplementary material for: Performance criteria for verbal autopsy-based systems to estimate national causes of death: development and application to the Indian Million Death Study
Source: BMC Med. 2014 Feb 4;12:21. doi: 10.1186/1741-7015-12-21 (PMC3912490; doi:10.1186/1741-7015-12-21)
Supplement: Additional file 4 — Mapping of Indian MDS cause of death categories to GBD 2010 and WHO-VA 2012. Describes the alignment of cause of death categories that was used for comparison across cause of death classification systems. [file 1741-7015-12-21-S4.pdf]

Additional file 4: Mapping of India MDS cause of death categories to GBD 2010 and WHO-VA 2012

Causes of death	MDS	WHO-VA 2012	GBD 2010
**Neonates (ages 0-28 days)**			
Prematurity & low birth weight	1C01	10.01	A.05.01
Birth asphyxia & birth trauma	1C02	10.02	A.05.02
Neonatal Infections	1A	10.03, 10.04	A.05.03
All other causes	1B,1D,2A-3A	10.05-10.06	A.05.04
Ill-defined conditions	4A	10.99	-
**Under 5 (ages 1-59 months)**			
Pneumonia	1A01	10.02, 10.03	A.02.03 TO A.02.05
Diarrhoeal diseases	1B01	10.04	A.02.01, A.02.02
Malaria	1B07	10.05	A.03.01
Other infections and parasitic diseases	1A (except 1A01), 1B (except 1B01, 1B07,1B09)	01.01, 01.03, 01.06-01.99, 10.04-10.05	A.01, A02 (Except A02.01-A02.05), A03 (except A.03.01), A05,A07
injuries	3A	12.01-12.99	C.01 TO C.04
All other causes	1C, 1D, 2A	02.01-08.01, 10.01-10.03, 10.06-10.99, 98	A05,A06,B.01 to B.10
Ill-defined conditions	1B09, 4A	99	-
**Adults (ages 5-69 years)**			
**Infections, parasitic diseases, maternal and nutritional conditions**			
Malaria	1H	01.05	A.03.01
Tuberculosis	1A	01.09	A.01.01
HIV/STI	1B,1C	01.03	A.01.02, A.07.01
Other infectious diseases	1A to 1L (exclude 1A to 1C, 1H)	01.01-01.02, 01.04, 01.06, 01.08, 01.10-01.99	A.01 to A.03 (exclude A.01.01, A.01.02, A.03.01) A.07(excl. A.07.01)
Maternal conditions	1M	09.01-09.99	A.04
Nutritional conditions	1O	03.01,03.02	A.06
**Chronic conditions**			
Cancer	2A	02.01-02.99	B.01
Heart disease	2G03	04.01	B.02.02
Stroke	2G04	04.02	B.02.03
Other CVD	2G (exclude 2G03,2G04)	04.03-04.04	B.02 (exclude B.02.02, B02.03)
Chronic respiratory diseases	2H	05.01-05.02	B.03
Cirrhosis of the liver	2J02	06.02	B.04
Other digestive diseases	2J (except 2J02)	06.01	B.05
Renal and other endocrine diseases	2B,2C,2K01	03.03, 07.01	B.08
Other chronic diseases	2D, 2F, 2K02 to 2L	07.01-09.99, 10.06, 98	B.06 to B10
**Injuries**			
Road traffic accidents	3A01	12.01	C.01.01
Suicides	3B01	12.08	C.03.01
Other injuries	3A (except 3A01),3B (except 3B01), 3C	12.02-12.07, 12.09-12.99	C.01 (except C.01.01), C.02, C.03 (except, C.03.01), C.04
**Ill-Defined conditions**			
Ill-defined conditions	1P, 4A	99	Redistributed*

Notes:

*In GBD, ill-defined conditions are removed from each disease category and redistributed among other diseases [27]. This table does not show redistributed deaths.
