# Supplementary material for: Performance criteria for verbal autopsy-based systems to estimate national causes of death: development and application to the Indian Million Death Study
Source: BMC Med. 2014 Feb 4;12:21. doi: 10.1186/1741-7015-12-21 (PMC3912490; doi:10.1186/1741-7015-12-21)
Supplement: Additional file 5 — (a) Numbers and proportion of physician agreement (where verbal autopsy records were assigned a final cause of death) by cause of death and stage of coding. Data based on adult deaths in the MDS. (b) Number of records, CSMF, and absolute CSMF error, by cause of death and stage of physician coding. Data based on adult deaths in the MDS. [file 1741-7015-12-21-S5.pdf]

Additional file 5a: Numbers and proportion of physician agreement by stage of coding

Cause of death	Initial Agreement	%	Reconciliation Agreement	%	Adjudication Agreement	%	Total
**Communicable**							
Malaria	1378	65.9%	323	15.4%	390	18.7%	2091
Tuberculosis	4620	80.4%	539	9.4%	588	10.2%	5747
HIV/STI	352	76.5%	51	11.1%	57	12.4%	460
Other infectious diseases	4272	60.8%	1336	19.0%	1424	20.3%	7032
Maternal conditions	634	63.4%	246	24.6%	120	12.0%	1000
Nutritional conditions	172	41.5%	112	27.1%	130	31.4%	414
**Non-communicable**							
Cancer	3735	66.1%	1023	18.1%	891	15.8%	5649
Heart diseases	5843	75.2%	949	12.2%	973	12.5%	7765
Stroke	3452	65.1%	999	18.8%	855	16.1%	5306
Other CVD	621	42.8%	383	26.4%	448	30.9%	1452
Chronic respiratory disease	4235	71.7%	793	13.4%	882	14.9%	5910
Cirrhosis of the liver	1695	55.2%	759	24.7%	615	20.0%	3069
Other digestive diseases	641	41.2%	426	27.4%	489	31.4%	1556
Renal and other endocrine diseases	1691	61.4%	545	19.8%	518	18.8%	2754
Other chronic diseases	941	45.1%	588	28.2%	557	26.7%	2086
**Injury**							
Road traffic accidents	1844	68.9%	549	20.5%	282	10.5%	2675
Suicides	2276	68.9%	670	20.3%	355	10.8%	3301
Other injuries	3091	60.5%	1191	23.3%	825	16.2%	5107
**Ill-defined conditions**	2074	47.5%	780	17.9%	1514	34.7%	4368
**All**	43567	64.3%	12262	18.1%	11913	17.6%	67742

Additional file 5b: Number of records and CSMF by cause of death and stage of physician coding

	Physician 1 initial code			Physician 2 initial code			Sum of initial codes			Aggregate (all stages**)			Final code	
	No.	CSMF	Error*	No.	CSMF	Error*	No.	CSMF	Error*	No.	CSMF	Error*	No.	CSMF
**Communicable**														
Malaria	1997	3.3%	-0.1%	1979	3.2%	-0.2%	3976	3.2%	-0.1%	5783	3.2%	-0.1%	2076	3.4%
Tuberculosis	5524	9.0%	-0.2%	5489	8.9%	-0.3%	11013	9.0%	-0.3%	13761	7.7%	-1.5%	5670	9.2%
HIV/STI	435	0.7%	0.0%	423	0.7%	0.0%	858	0.7%	0.0%	1107	0.6%	-0.1%	434	0.7%
Other infectious diseases	6721	11.0%	-0.3%	6547	10.7%	-0.6%	13268	10.8%	-0.5%	19970	11.2%	-0.1%	6918	11.3%
Maternal conditions	846	1.4%	-0.1%	847	1.4%	-0.1%	1693	1.4%	-0.1%	2695	1.5%	0.1%	889	1.4%
Nutritional conditions	409	0.7%	0.1%	403	0.7%	0.0%	812	0.7%	0.0%	1443	0.8%	0.2%	377	0.6%
**Non-communicable**														
Cancer	5331	8.7%	-0.2%	5320	8.7%	-0.2%	10651	8.7%	-0.2%	14967	8.4%	-0.5%	5449	8.9%
Heart Diseases	7278	11.9%	-0.4%	7370	12.0%	-0.2%	14648	11.9%	-0.3%	19000	10.7%	-1.6%	7513	12.2%
Stroke (cerebrovascular disease)	4366	7.1%	-0.2%	4277	7.0%	-0.4%	8643	7.0%	-0.3%	12457	7.0%	-0.4%	4505	7.3%
Other CVD	726	1.2%	0.1%	744	1.2%	0.1%	1470	1.2%	0.1%	2541	1.4%	0.3%	665	1.1%
Chronic Respiratory diseases	5506	9.0%	0.1%	5563	9.1%	0.2%	11069	9.0%	0.1%	14979	8.4%	-0.5%	5449	8.9%
Cirrhosis of the Liver	2397	3.9%	-0.1%	2474	4.0%	0.0%	4871	4.0%	0.0%	7816	4.4%	0.4%	2448	4.0%
Other digestive diseases	1415	2.3%	0.0%	1406	2.3%	0.0%	2821	2.3%	0.0%	5046	2.8%	0.6%	1398	2.3%
Renal and other endocrine diseases	2413	3.9%	-0.1%	2431	4.0%	-0.1%	4844	3.9%	-0.1%	7273	4.1%	0.0%	2479	4.0%
Other chronic diseases	1722	2.8%	0.1%	1730	2.8%	0.1%	3452	2.8%	0.1%	6128	3.4%	0.7%	1662	2.7%
**Injuries**														
Road traffic accidents	1986	3.2%	-0.1%	1980	3.2%	-0.1%	3966	3.2%	-0.1%	5525	3.1%	-0.2%	2044	3.3%
Suicides	2564	4.2%	-0.1%	2480	4.0%	-0.2%	5044	4.1%	-0.2%	6986	3.9%	-0.4%	2632	4.3%
Other injuries	4209	6.9%	0.0%	4289	7.0%	0.1%	8498	6.9%	0.0%	12859	7.2%	0.3%	4225	6.9%
**Ill-defined conditions**	5470	8.9%	1.6%	5591	9.1%	1.7%	11061	9.0%	1.7%	17814	10.0%	2.6%	4520	7.4%
**All**	**61315**	**100%**	**3.9%**	**61343**	**100%**	**4.8%**	**122658**	**100%**	**4.2%**	**178150**	**100%**	**10.58%**	**61353**	**100%**

*CSMF absolute error compared to final assigned codes

** Including initial coding, reconciliation, and adjudication
